# Flexible 3D Plasmonic Web Enables Remote Surface Enhanced Raman Spectroscopy

**DOI:** 10.1002/advs.202402192

**Published:** 2024-04-06

**Authors:** Erika Rodríguez‐Sevilla, Jonathan Ulises Álvarez‐Martínez, Rigoberto Castro‐Beltrán, Eden Morales‐Narváez

**Affiliations:** ^1^ Centro de Investigaciones en Óptica A. C. Loma del Bosque 115, Lomas del Campestre León Guanajuato 37150 México; ^2^ Departamento de Ingeniería Física División de Ciencias e Ingenierías Universidad de Guanajuato Loma del Bosque 103, Lomas del Campestre León Guanajuato 37150 México; ^3^ Biophotonic Nanosensors Laboratory Centro de Física Aplicada y Tecnología Avanzada (CFATA) Universidad Nacional Autónoma de México (UNAM) Boulevard Juriquilla 3001 Querétaro 76230 México

**Keywords:** 2D materials, flexible photonics, green technologies, nanophotonics, plasmonic field transport, ultrasensitive sensors

## Abstract

Nanoplasmonic materials concentrate light in specific regions of dramatic electromagnetic enhancement: hot spots. Such regions can be employed to perform single molecule detection via surface‐enhanced Raman spectroscopy. However, this phenomenon is challenging since hot spots are expected to be highly intense/abundant and positioning of molecules within such hot spots is crucial to manage with ultrasensitive SERS. Herein, it is discovered that a 3D plasmonic web embedded within a biohybrid (3D‐POWER) exhibits plasmonic transmission, spontaneously absorbs the analyte, and meets these so much needed criteria in ultrasensitive SERS. 3D‐POWER is built with nanopaper and self‐assembled layers of graphene oxide and gold nanorods. According to in silico experiments, 3D‐POWER captures light in a small region and performs plasmonic field transmission in a surrounding volume, thereby activating a plasmonic web throughout the simulated volume. The study also provides experimental evidence supporting the plasmonic field transport ability of 3D power, which operates as a SERS signal carrier (even beyond the apparatus field of view), and the ultrasensitive behavior of this ecofriendly and flexible material facilitating yoctomolar limit of detection. Besides, 3D‐POWER is proven useful in food and biofluids analysis. It is foreseen that 3D‐POWER can be employed as a valuable platform in (bio)analytical applications.

## Graphical abstract

A flexible material offering a plasmonic network is reported. The hot spots of the network are not only locally but also remotely active, even beyond the excitation region. The plasmonic network also carries surface enhanced raman scattering signals, offering ultrasensitive detection capabilities, which can reveal the presence of harmful compounds in food, as well as metabolites in biofluids.

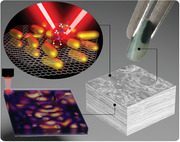

## 1 Introduction

Surface enhanced Raman Spectroscopy (SERS) provides valuable information on the structural fingerprint of molecules at extremely low concentrations in a label‐free, non‐destructive, and high‐throughput fashion. Hence, SERS is finding a myriad of unprecedented applications in cultural heritage, chemistry, catalysis, biology, biomedicine, diagnostics, nanotechnology, materials characterization, energy storage/conversion, as well as in food, environmental, and measurement science.^[^ ^1^, ^2^, ^3^, ^4^, ^5^ ^]^

SERS signals are typically unfolded by means of the interaction of a molecule with specific regions of dramatic electromagnetic enhancement (“hot spots”) occurring in the proximity of metallic nanostructures, such as plasmonic nanomaterials. Hence, this phenomenon provides the capability to detect the analyte at the single molecule (SM) level. To this end, hot spots are expected to facilitate high electromagnetic enhancement factors (e.g. from 10^5^ to 10^10^).^[^ ^5^ ^]^ Hence, several SERS substrates incorporating this class of hot spots have been proposed, including nanoparticles aggregates/junctions, sharp edges, sharp nanoroughness, and nanoparticle gaps and crevices,^[^ ^6^ ^]^ where rationally designed nanogaps have been reported to be highly reproducible,^[^ ^7^ ^]^ but hot spots should be carefully designed according to the size of the target molecule.^[^ ^8^, ^9^, ^10^, ^11^, ^12^ ^]^ In practice, single molecule SERS is generally carried out with limits of detection from the nanomolar to the picomolar range.^[^ ^13^, ^14^, ^15^ ^]^ Therefore, the capability to increase hot spot allocation is highly desirable in ultrasensitive or single molecule SERS. In addition, accurate delivery and SERS signal collection of a single molecule within a hot spot is generally challenging and/or time‐consuming.^[^ ^5^, ^7^ ^]^ To circumvent this issue, approaches of surface engineering that lead to highly controllable surface wettability have been established, and they preconcentrate the analyte to achieve limits of detection from the femto to the attomolar range.^[^ ^14^, ^16^ ^]^ Nevertheless, the fabrication of these substrates often requires complex and expensive infrastructure, such as nano/microfabrication facilities.

Herein, we report that a 3D plasmonic web embedded within a biohybrid (3D‐POWER), exhibits plasmonic field transport capabilities, and thus its hot spots are not only locally but also remotely active to offer ultrasensitive SERS with a limit of detection up to the yoctomolar range. In fact, we discovered that this plasmonic network can report the SERS spectrum of a molecule located beyond both the apparatus laser beam and the field of view when the analyte is incorporated within the active sites of 3D‐POWER, which was supported by in silico and experimental approaches. 3D‐POWER was architected with nanopaper and self‐assembled layers of graphene oxide and gold nanorods via wet chemistry. We also demonstrated that 3D‐POWER is an advantageous platform for the analysis of real samples of food (corn, apple, orange, lettuce, tomato and mango) and biofluids such as sweat and sebum.

## 2 Results and Discussion

### 2.1 Fabrication and Characterization

**Scheme 1:**
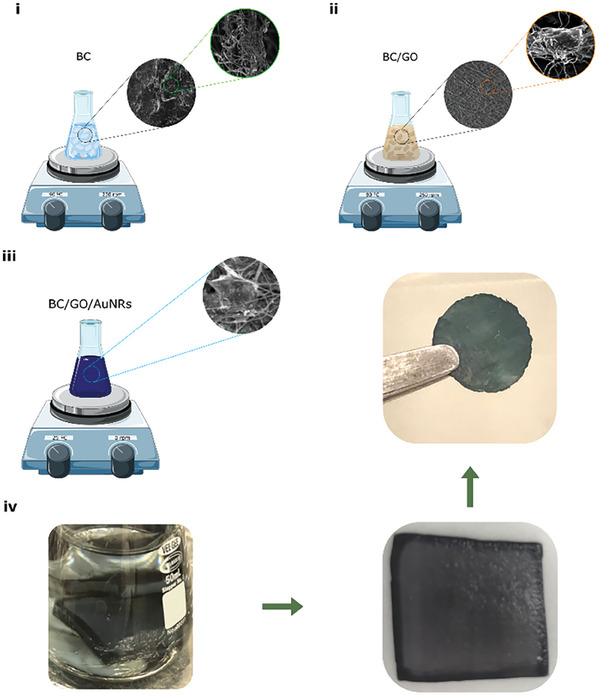
3D‐POWER fabrication process. i) BC is washed with ultrapure water. ii) BC is added in a GO suspension. GO is spontaneously self‐assembled by layers within BC. iii) BC/GO is added in a suspension of AuNRs. AuNRs are absorbed within BC/GO in this process. iv) 3D‐POWER is washed and dried. Full details on this synthesis process are described in the Experimental Section. Table S1 (Supporting Information) offers details on the (nano)metrology and physicochemical features of each component of 3D‐POWER, see the Supporting Information.

3D‐POWER was readily fabricated by incorporating graphene oxide (average lateral size of ≈500 nm) and gold nanorods (average length ≈50 ± 10 nm, average diameter ≈14 ± 3 nm) within bacterial nanocellulose (BC) in the liquid phase, see **Scheme** **1** and Schemes S1 and S2 (Supporting Information). Full details on the synthesis of 3D‐POWER are described in the experimental section. Graphene oxide (GO) was self‐assembled within BC via hydrogen bonding and the high wettability of BC. The (nano)metrology and physicochemical properties (such as the surface chemistry) of each component, which led to the resulting self‐assembled composite, are summarized in Table S1 (Supporting Information). On the one hand, according to theoretical models, gold nanorods (AuNRs) exhibit a superior SERS enhancement when compared with gold nanospheres.^[^ ^17^ ^]^ On the other hand, 3D SERS substrates incorporating GO are a suitable alternative to address common problems of SERS such as sample fluorescence and sample/substrate degradation. GO also facilitates the attachment of organic compounds by means of *π–π* stacking interactions, thereby leading to a charge‐transferring phenomenon that enhances SERS behavior. In addition, GO masks the SERS signature of cellulose (which can be considered noise, see Figure S1, Supporting Information) produced by BC in nanocellulose‐based SERS substrates.^[^ ^18^ ^]^ In this context, we reasoned that a 3D SERS substrate based on AuNRs and GO could be advantageous.

**Figure 1:**
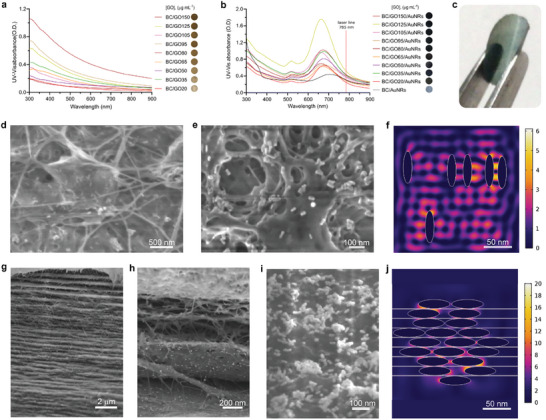
Characterization of the explored materials. UV–vis absorbance spectra and pictures of a) graphene oxide decorated nanopapers (BC/GO) and b) graphene oxide and gold nanorods decorated nanopapers (BC/GO/AuNRs). c) Picture showing the flexible character of the composite. d,e) SEM micrographs of the surface of BC/GO35/AuNRs. f) In silico experiment depicting the electromagnetic enhancement onto the surface of BC/GO35/AuNRs. g–i) SEM micrographs of the transversal view of BC/GO35/AuNRs. j) In silico experiment depicting the electromagnetic enhancement within the inner part of BC/GO35/AuNRs.

Firstly, we explored the UV‐VIS spectroscopy of different composite materials manufactured with several concentrations of graphene oxide (from 20 to 150 µg mL^‐1^, see **Figure** **1**a) and a constant concentration of AuNRs (0.7 mm), see Figure 1b. The flexible character of the resulting composites is exemplified in Figure 1c. Existing literature suggests that the intensity of the optical density of 3D SERS substrates based on BC and plasmonic nanoparticles can be related to the density of the nanoparticles embedded within the substrate. Hence, high optical density of BC‐based SERS substrates can be due to the plasmonic coupling provoked by a high density of plasmonic nanoparticles leading to a high density of hot spots.^[^ ^18^ ^]^ Actually, because of the surface chemistry of GO combined with BC, see Table S1 (Supporting Information), GO facilitated the incorporation of a high density of AuNRs within BC (e.g. 493 ± 72 particles per µm^2^), when compared with BC embedding AuNRs in the absence of GO (105 ± 19 particles per µm^2^), see Table S2 (Supporting Information). According to our results summarized in Table S2 (Supporting Information) and Figure 1b, we achieved the highest optical densities using GO concentrations of 35, 80, and 125 µg mL^−1^ in the fabrication of the composites, from now and then termed as BC/GO35/AuNRs, BC/GO80/AuNRs and BC/GO125/AuNRs, respectively. Therefore, given the hot spot density enabled by these biohybrids, these composites were chosen for further investigation.

We analyzed the morphology and particle density of the selected biohybrids using scanning electron microscopy (SEM), see Figure 1d,e and Figure S2 (Supporting Information). Remarkably, comparing micrographs of transversal SEM views (frontal planes) of those composites fabricated in the presence and absence of GO (BC/AuNRs), we observed that GO and AuNRs were self‐assembled in layers within BC, see Figure 1g,i and Figure S3 (Supporting Information). The surface (horizontal plane) of BC/AuNRs had a density of 105 ± 19 particles per µm^2^, whereas the surface of BC/GO35/AuNRs, BC/GO80/AuNRs, and BC/GO125/AuNRs, exhibited a density of 150 ± 14, 207 ± 37, and 493 ± 72 particles per µm^2^, respectively, see Table S3 (Supporting Information). However, micrographs of transversal views of the 3D biohybrids revealed that the inner part of the composites exhibited a different particle density. The particle density in the transversal SEM micrographs of BC/AuNRs was of 358 ± 29 particles per µm^2^, whereas those of BC/GO35/AuNRs, BC/GO80/AuNRs, and BC/GO125/AuNRs, were of 497 ± 25, 402 ± 61, and 270 ± 37 particles per µm^2^, respectively. Figures S2 and S3 (Supporting Information), depict the corresponding micrographs. Hence, we concluded that, in the final architecture of 3D‐POWER, medium or high concentrations of GO (e.g., from 80 µg mL^‐1^) promote the adsorption of AuNRs, whereas low concentrations of GO (≈35 µg mL^‐1^) facilitate the absorption of AuNRs, which is summarized in Table S3 (Supporting Information). Hence, in terms of the architecture of the explored biohybrid, GO assists impregnation of AuNRs and self‐organization of GO/AuNRs layers in 3D‐POWER, see Figure 1d,e,g,i.

Considering the resulting particle density and the optical properties of the employed materials, we performed in silico approaches revealing the electromagnetic enhancement enabled by 3D‐POWER, particularly with the biohybrid offering the highest density of hot spots in the transversal micrographs, that is, BC/GO35/AuNRs, see Figure 1f,j. All the details related to the in silico experiments are given in the Experimental Section, which allowed for the theoretical determination of the electromagnetic field enhancement factors; *γ*, calculated as |*E_loc_* |^4^ */*|*E_0_* |^4^, where *E_0_* is the electric field of the incident light, while *E_loc_* is the localized electric field at the respective hot spot. A simulation of the plasmonic activity in a horizontal plane of 3D‐POWER demonstrated that the biohybrid displays a network of hot spots with *γ* values ranging from 25 to 1376, see Figure 1f and Figure S4 (Supporting Information), whereas a frontal plane of 3D‐POWER gave rise to *γ* values from 130 to 42522, see Figure 1j and Figure S5 (Supporting Information). Although horizontal planes of BC/GO35/AuNRs displayed similar mean values of *γ* when compared with horizontal planes of BC/AuNRs, see Table S4 (Supporting Information), frontal planes of BC/GO35/AuNRs displayed mean values of *γ* much higher than those shown by BC/AuNRs (c.a. 17 times higher), see Figures S6 and S7 and Table S4 (Supporting Information). Hence, the presence of GO in the biohybrid also led to the improvement of *γ*.

### 2.2 Surface Enhanced Raman Spectroscopy Behavior

**Figure 2:**
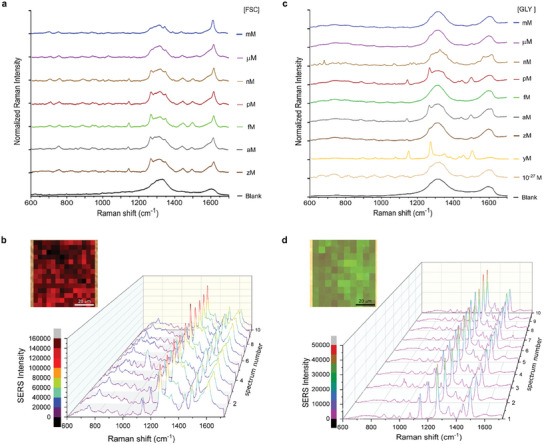
3D‐POWER as a SERS substrate. a) SERS spectra of 3D‐POWER impregnated with FITC (FSC) at different concentrations (from mm to zm). b) Representative SERS spectra and SERS mapping (relative intensity of the peak at 1616 cm^−1^) offered by 3D‐POWER impregnated with [FSC] = 10^−21^ m (zm). c) SERS spectra of 3D‐POWER impregnated with Glyphosate (GLY) at different concentrations (from mm to ym). d) Representative SERS spectra and SERS mapping (relative intensity of the peak at 1500 cm^−1^) offered by 3D‐POWER impregnated with [GLY] = 10^−24^ m (ym). Each spectrum represents the average of at least one hundred spectra recorded onto an area of 2500 µm^2^ of 3D‐POWER. All Raman measurements were performed using an excitation wavelength 785 nm, power, 0.08 mW, and exposure time, 2 s, size of the spot = 1.2 µm, and number of acquisitions, 10.

After the evaluation of the SERS performance of the selected substrates (BC/AuNRs, BC/GO35/AuNRs, BC/GO80/AuNRs BC/GO125/AuNRs), see section Selection of the optimal SERS substrate in the Supporting Information, BC/GO35/AuNRs was selected as the optimal one. We then explored the SERS performance of 3D‐POWER by carrying out an overnight incubation of millimetric circles (diameter, 6 mm) of the biohybrid (BC/GO35/AuNRs) inside a microtube containing 1 mL of the analyte at different concentrations, blank samples were also considered in this series of experiments as a negative control. Fluorescein (FSC), CAS 2321‐07‐5, and glyphosate (GLY), CAS 287399‐31‐9, were employed as model analytes. Surprisingly, using 3D‐POWER as a SERS substrate, we experimentally realized that we were able to detect extremely low concentrations of the model analytes. **Figure** **2**a,b shows the SERS performance of 3D‐POWER upon FSC incubation, where we detected FSC concentrations at the zeptomolar range, see Figure S12 and Table S5 (Supporting Information) detailing the number of FSC molecules. Besides, Figure S9 and Table S8 (Supporting Information) summarize the observed vibrational bands.

The repeatability of the fabrication of the biohybrid was explored by manufacturing 5 batches of BC/GO35/AuNRs, see Figure S8 and Table S7 (Supporting Information). Using FSC as a model analyte, we also performed an analysis of the repeatability and signal intensity robustness across different batches. Although the Raman intensity at the Raman shift of 1616 cm^−1^ was not particularly consistent across batches, see Table S7 (Supporting Information), in general, the Raman signature of FSC was observable up to the zeptomolar range across different batches of the explored biohybrid, see Figure S11 (Supporting Information). Hence, the sensitivity of the SERS observed in this research can be considered repeatable. In addition, a robust signal intensity at the Raman shift of 1616 cm^−1^ accounted for at least 5500 counts even when analyzing FSC concentrated at the zeptomolar range, see Table S7 and Figure S12 (Supporting Information). Figure 2c,d displays the SERS performance of 3D‐POWER upon GLY impregnation, where GLY was detected at the yoctomolar level, see Table S10 (Supporting Information), estimating the number of molecules contained in the GLY samples and eventually reaching the single molecule level. Figures S12–S14 (Supporting Information) display experimental examples of the high quality of the spectra that can be acquired using 3D‐POWER. Table S11 (Supporting Information) shows the respective vibrational bands. In spite of the ultrasensitive character of 3D‐POWER, as a shortcoming to be addressed in future works, the explored SERS substrate did not show a quantitative behavior, see Figures S11‐S13 (Supporting Information). Hence, the proposed SERS substrate is currently limited to qualitative detection.

The model analytes can also interact with 3D‐POWER to yield a charge‐transfer enhancement of Raman scattering. FSC can interact with 3D‐POWER via *π–π* stacking, whereas GLY interacts with the surface chemistry of 3D‐POWER by means of the phosphonate group in P‐O^−^, P═O^−^ and P‐C‐N‐H bonds, which is stabilized by hydrogen interactions.^[^ ^19^ ^]^ However, given the molecular flexibility of GLY (due to its single bonds, see Figure S14, Supporting Information), a concentration‐dependent SERS fingerprint was observed for this analyte. Hence, the surface coverage of GLY onto 3D‐POWER may modulate the orientation of GLY yielding such a concentration‐dependent spectroscopic behavior,^[^ ^20^ ^]^ where the lowest concentrations of GLY allowed for the best observations of a highly detailed Raman signature of GLY by means of 3D‐POWER, see Figures S14 and S15 (Supporting Information), particularly, displaying clearly defined vibrational bands associated to the phosphonate group, see Table S11 (Supporting Information). In the analysis of FSC, we observed an extraordinary analytical enhancement factor of up to c.a. 8×10^16^, see **Table** **1** and Table S9 (Supporting Information). In the analysis of GLY, we observed an unprecedented analytical enhancement factor of up to c.a. 6×10^21^, see Table 1, Table S12 (Supporting Information) and Determination of the Analytical Enhancement Factor Sub‐Section in the Experimental Section. (In this context, chemical enhancement of Raman spectroscopy produced by graphene‐related materials should not be underestimated, which can be as large as ≈10^3^).^[^ ^21^ ^]^ Intrigued by these results, we hypothesized that such ultrasensitive behavior can only be achieved by means of a remote SERS effect enabled by a 3D plasmonic network.

**Table 1:** Comparison of the analytical enhancement factor.

Analyte	SERS Substrate	Raman shift [cm^−1^]	Concentration	AEF	Reference
FSC	3D‐POWER	1616	10^−21^ m	8×10^16^	This work
	rGOS‐AuNPs films	1181.8	10^−7^ m	8×10^5^	[13]
FSC	MoS_2_@Au/Ag hybrid GF	1183	10^−12^ m	5×10^6^	[15]
Glyphosate	3D‐POWER	1500	10^−24^ m	6×10^21^	This work
	Ag/Cu‐grid	1440	5×10^−6^ m	6×10^5^	[16]
	Si/TiO_2_/Ag	NR	6×10^−10^ m	NR	[17]
	TiO_2_ NTs/AgNPs‐rGO	920	2×10^−8^ m	NR	[18]
	BiOCl‐BiOBr@Pt/Au	1011	10^−10^ m	10^6^	[19]

AEF, Analytical Enhancement Factor. Fluorescein, FSC. Ag, silver. MoS_2_, Molybdenum disulfide; Au, gold. GF, glass fiber. rGOS, reduced graphene oxide by Graphene Sypermarket (Ronkonkoma). Cu‐grid, copper discs with a fine mesh. Si, silicon. TiO2, titania. TiO2 NTs, titania nanotubes. AgNPs, silver nanoparticles. rGO, reduced graphene oxide. BiOCl‐BiOBr, bismuth oxyhalides. Pt, platinum. NR, not reported.

### 2.3 Plasmonic Transport and Remote SERS

Typically, remote SERS involves plasmonic waveguides to excite/detect analytes remotely, which is generally facilitated by metallic nanowires, semiconductors, organic submicrowires, and organic (submicro)tubes.^[^ ^22^, ^23^ ^]^ In addition, nanoantennas consisting of plasmonic nanoparticles deposited on a metal film spaced by ultrathin dielectric nanogaps have been reported to capture light, behave as plasmonic waveguides and transmit SERS signals.^[^ ^24^ ^]^ Dissolved oxygen removal also facilitates long‐range plasmonic field transmission, which leads to a zeptomolar sensitivity via remote SERS.^[^ ^13^ ^]^ Considering this context, we decided to explore the plasmonic transport character of 3D‐POWER.

We performed in silico experiments to investigate the light transport capability of the materials composing 3D‐POWER, all of them considered the same surface area and electromagnetic conditions. Further details on the performed simulations and their workflow are described in the Experimental Section (In silico experiments Sub‐Section) and the Supporting Information. The employed constants (permittivity, permeability, and conductivity) are detailed in Table S13 (Supporting Information). Light propagation simulations were performed by establishing the pumped region (c.a. 10×10^3^ nm^2^) at the upper right corner of the simulated area of the corresponding material, see an example in Figure S16e,f (Supporting Information). As observed in Figure S16a (Supporting Information), light propagation simulations of bare BC exhibited negligible light transport capabilities. However, light‐guiding properties were noticed in the simulated area of BC/GO, demonstrating that the presence of self‐assembled GO in BC offers light propagation properties, see Figure S16b (Supporting Information). Simulations of the light‐BC/AuNRs interaction, where AuNRs were randomly oriented, exhibited electromagnetic field confinement and light propagation by inner scattering in the explored material, see Figure S16c (Supporting Information). In silico experiments also demonstrated that the plasmonic intensity and light‐guiding properties are superior in the plasmonic web facilitated by BC/GO/AuNRs, when compared with BC/GO or BC/AuNRs, see Figure S16d (Supporting Information). Using light excitation perpendicular to the self‐assembled layers of 3D‐POWER, 3D simulations revealed that BC/GO/AuNRs was able to capture light in a small region (5×10^5^ nm^3^) and perform plasmonic field transmission, thereby generating a plasmonic web throughout the simulated volume (8×10^6^ nm^3^ and 3.2×10^7^ nm^3^, respectively), see Figures S16e,f and S17a,b (Supporting Information). However, in silico experiments also suggest that under an excitation that is parallel to the self‐assembled layers, 3D‐POWER also offers highly efficient transport of surface plasmon polaritons as observed in Figure S17c (Supporting Information). It is worth discussing that typically plasmonic waveguides operate at distances over tens of microns,^[^ ^22^ ^]^ however long‐range/millimetric propagation is also feasible by means of plasmonic waveguides involving dielectric and metallic materials.^[^ ^25^ ^]^

We also found experimental evidence of the plasmonic filed transport capability of 3D‐POWER. In the aforementioned system of nanoantennas (deposited on a metal film spaced by ultrathin dielectric nanogaps), surface plasmon polaritons travel between nanoparticles and remotely light transmitting antennas, where dark field images reveal such a remote phenomenon.^[^ ^24^ ^]^ Likewise, we also recorded dark field images of 3D‐POWER, where probably nanoparticles beyond the laser spot (excitation wavelength, 785 nm) showed bright emissions due to an excitation via transmitted surface plasmon polaritons, see Figure S18 (Supporting Information). Existing literature suggests that this kind of surface plasmon polaritons could operate as a non‐interfering information carrier, particularly transmitting SERS signals. Moreover, the same literature reported that the orientation of plasmonic nanoparticles did not affect remote spectroscopy performance significantly.^[^ ^24^ ^]^

**Figure 3:**
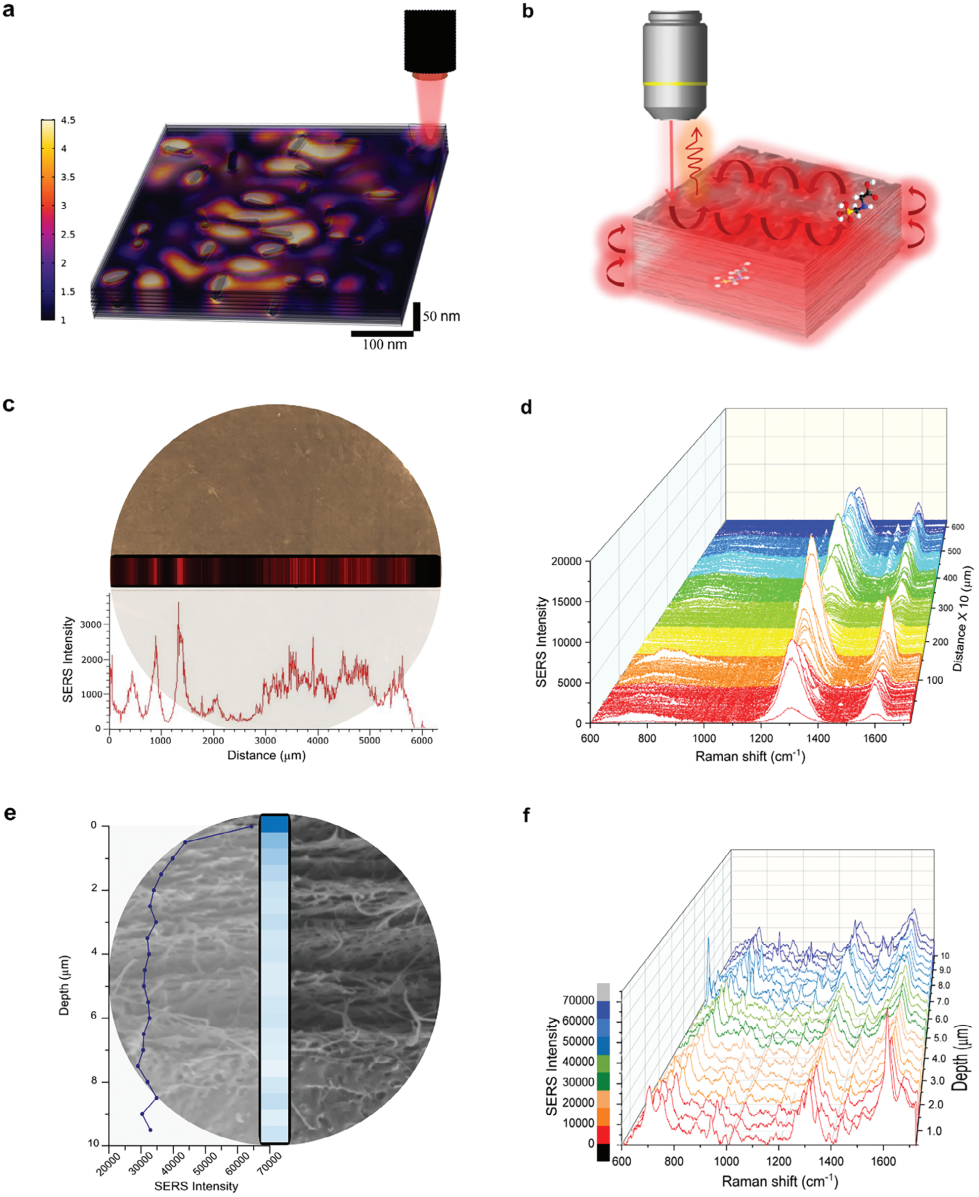
3D‐POWER as a platform that transports plasmonic fields and transmits SERS signals. a) In silico experiment demonstrating highly efficient transport of surface plasmon polaritons in 3D‐POWER. b) Schematic representation of the behavior of 3D‐POWER: the plasmonic web not only transports plasmonic fields, but also transmits SERS signals. c,d) Raman mapping (x–y plane) at 10 µm per step. Remote detection of c.a. 10 FSC molecules throughout the diameter of a millimetric piece of 3D‐POWER. Signal intensities shown in (c) correspond to the intensity at the Raman shift of 1500 cm^−1^. e,f) Raman mapping (z‐axis) at 0.5 µm per step. Remote detection of c.a. 10 FSC molecules throughout the z‐axis of a millimetric piece of 3D‐POWER. Signal intensities depicted in (e) correspond to the intensity at the Raman shift of 1500 cm^−1^.

We also performed a series of experiments to prove the behavior of the biohybrid as a plasmonic web carrying SERS signals. We drop casted 0.5 µL of FSC concentrated at 10^−9^ m (c.a. 9.5×10^8^ molecules) onto the extreme part of a piece of 54 mm^2^ of the biohybrid and, given a capillary driven flow, the sample run c.a. 27 mm^2^. We then performed a Raman mapping in the frontier of the substrate that was not reached by the sample. Surprisingly, although the analyte was not deposited onto the explored zone, such a mapping revealed that a remote SERS phenomenon was observable at least throughout 700 µm as depicted in Figure S19 (Supporting Information). We then drop casted 0.1 µL of FSC concentrated at 10^−16^ M (c.a. 10 molecules) onto the center of a circular piece of 6 mm of 3D‐POWER, and a Raman mapping with a resolution of 500 µm per step was recorded throughout the diameter of the substrate see Figure S20 (Supporting Information). In spite of the extremely low number of molecules deposited onto the surface of the substrate, SERS spectra were revealed by means of 3D‐POWER. We then repeated this experiment but increased the resolution of the Raman mapping at 10 µm per step. Although the recorded SERS intensity was not particularly homogenous, which is expected in single molecule detection,^[^ ^26^ ^]^ we confirmed the remote detection of c.a. 10 FSC molecules throughout the diameter of a millimetric piece of 3D‐POWER, see **Figure** **3**c,d. To explore the 3D character of the resulting plasmonic web, in a similar experiment with c.a. 10 molecules of FSC deposited onto the biohybrid, we also performed a vertical Raman mapping (in the z‐axis of 3D‐POWER) with a resolution of 0.5 µm per step. Figure 3e,f shows that the plasmonic web reporting SERS signals not only operate throughout a single plane of the SERS substrate, but SERS signals are also detectable throughout the vertical axis (ca. 10 µm) of 3D‐POWER. Considering the above discussed in silico experiments and this series of experiments, we concluded that the explored plasmonic web not only transports plasmonic fields, see Figure 3a and Figures S16 and S17 (Supporting Information), but also carries SERS signals, which can be captured by the collector of the employed Raman apparatus; however, the analyte is not particularly located within the laser spot, but embedded within the plasmonic web enabled by 3D‐power, see a schematic representation of this concept in Figure 3b. Actually, the aforementioned qualitative analytical behavior could be due to this operating principle of 3D‐POWER, as 3D‐POWER virtually enables the detection of a molecule embedded within the plasmonic network, whereas conventional SERS only facilitates the detection of those molecules located in the respective laser beam.

### 2.4 Food and Biofluids Analysis

**Figure 4:**
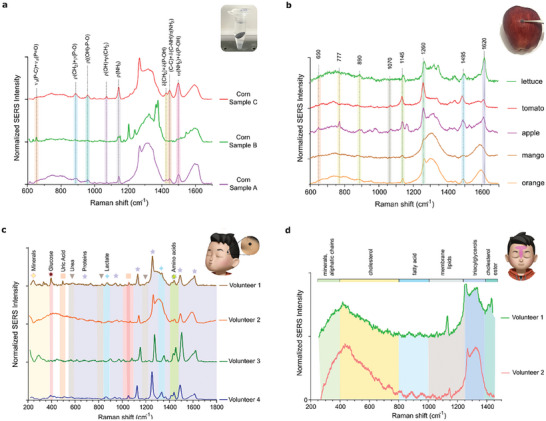
Food and biofluids analysis facilitated by 3D‐POWER. a) Corn samples analysis. b) Vegetables and fruits analysis. c) Sweat analysis. d) Sebum analysis.

Eventually, we employed 3D‐POWER to perform the SERS analysis of food and bodily fluids (no spiking process was performed in this series of experiments). The experimental procedures are described in the Experimental Section. We analyzed three different corn samples; corn sample A, white corn (commercially available); corn sample B, blue corn (commercially available); and corn sample C, white corn (sample taken in a local field). Corn powder samples were diluted in water HPLC grade (from 1 mg mL^−1^ to lower concentrations, respectively), and different pieces of 3D‐POWER were incubated overnight within these samples. Corn samples A and C from white corn exhibited Raman bands which can be potentially ascribed to the presence of organophosphates pesticides such as isofenphos‐methyl,^[^ ^27^ ^]^ benzimidazole,^[^ ^28^ ^]^ and glyphosate;^[^ ^29^, ^30^, ^31^, ^32^ ^]^ whereas the corn sample B was observed not to present Raman fingerprints associated with these pesticides, see **Figure** **4**a and Figures S21 and S22 (Supporting Information). Hence, corn sample B was probably grown in the absence of the aforementioned pesticides. 3D‐POWER substrates were also rubbed into the surface of vegetables (lettuce and tomato) and fruits (apple, mango, orange). The lettuce surface smear exhibited Raman fingerprints which can be potentially attributed to the presence of nylon (an agro‐textile that is used as a mesh to protect crops from insects),^[^ ^33^, ^34^, ^35^ ^]^ and some agents with antibiotic activity, including thiram^[^ ^36^ ^]^ and thiabendazole^[^ ^37^ ^]^ see Figure 4b, Figure S23a and Table S14 (Supporting Information). The tomato surface smear displayed Raman spectral features which can be potentially associated with carotenes,^[^ ^38^ ^]^ xylene (a pesticide carrier),^[^ ^39^ ^]^ an some biocides such as acetamiprid,^[^ ^39^ ^]^ triazine and diphenyl ether,^[^ ^38^, ^39^ ^]^ see Figure 4b, Figure S23b and Table S14 (Supporting Information). The SERS analysis of the apple surface smear revealed the presence of Raman signatures which could be potentially related to harmful agents such as organophosphorus compounds,^[^ ^39^ ^]^ oxadiazine,^[^ ^39^ ^]^ glyphosate,^[^ ^39^ ^]^ thiram,^[^ ^40^ ^]^ acetamiprid,^[^ ^39^ ^]^ diquat,^[^ ^39^ ^]^ aryloxyalkanoic acid,^[^ ^39^ ^]^ thiabendazole,^[^ ^37^ ^]^ strobilurin, and triazole,^[^ ^39^ ^]^ see Figure 4b, Figure S23c and Table S14 (Supporting Information). The mango surface smear also exhibited Raman fingerprints which can be potentially ascribed to harmful compounds such as triazole,^[^ ^39^ ^]^ atrazine,^[^ ^39^ ^]^ glyphosate and organophosphorus compounds,^[^ ^29^, ^39^ ^]^ see Figure 4b, Figure S23d and Table S14 (Supporting Information). The orange surface smear also displayed Raman spectral features which can be potentially attributed to pesticides such as phoxim,^[^ ^41^ ^]^ thiram,^[^ ^41^ ^]^ melathion,^[^ ^41^ ^]^ and glyphosate,^[^ ^29^ ^]^ see Figure 4b, Figure S23e and Table S14 (Supporting Information). Additionally, circular pieces of 3D‐POWER were stuck onto the temple of 4 sweating volunteers for 5 min in order to impregnate the SERS substrate with their sweat samples.  As depicted in Figure 4c and Table S15 (Supporting Information), Raman signatures potentially related to different metabolites, including uric acid,^[^ ^42^ ^]^ lactic acid/lactate,^[^ ^43^ ^]^ glucose,^[^ ^44^ ^]^ tyrosine,^[^ ^45^ ^]^ urea,^[^ ^45^ ^]^ arginine,^[^ ^46^ ^]^ histamine,^[^ ^47^ ^]^ and amino acids,^[^ ^48^ ^]^ were detected in the sweat of the volunteers by means of 3D‐POWER. T zone face smears of two volunteers were also carried out in order to impregnate circular pieces of 3D‐POWER with sebum. In this proof of concept, the SERS analysis facilitated by 3D‐POWER revealed Raman fingerprints potentially related to aliphatic chains,^[^ ^49^ ^]^ cholesterol, squalene,^[^ ^49^, ^50^ ^]^ cholesterol ester,^[^ ^51^ ^]^ fatty acids,^[^ ^49^ ^]^ membrane lipids,^[^ ^51^ ^]^ triacylglycerols,^[^ ^51^ ^]^ and vitamin E,^[^ ^50^ ^]^ see Figure 4d and Table S16 (Supporting Information).

Although the aforementioned Raman assignations could be potentially useful for the determination of hazardous compounds or metabolites, a detailed research comparing this proof of concept with other analytical techniques should be performed in order to validate such results and establish reliable applications. The performance of the 3D web of hot spots enabled by 3D‐POWER was investigated using small molecules, but given the 3D flexible nature of the substrate, we speculate that 3D‐POWER might be useful in the analysis of macromolecules (with size of few nanometers); however, this should be systematically investigated. SERS analysis of real samples is a complex task, where SERS substrates can be employed in a label‐free or a label‐based mode. Label‐based approaches are ideal to detect a target molecule in a highly specific way, avoiding complexity from non‐specific signals (coming from non‐target molecules), but they require reporter molecules. In contrast, label‐free detection enables reagent‐less analysis as well as spectral diagnostics; however, in order to address complexity from non‐specific signals, accurate SERS‐based label‐free detection requires strict controls, significant statistics, and massive data analysis, which can be implemented using artificial intelligence.^[^ ^52^ ^]^

## 3 Conclusion

We manufactured and studied the photonic properties of a 3D flexible composite material made of self‐assembled layers of GO decorated with AuNRs embedded within BC. In silico approaches and experimental evidence suggest that the hot spots of 3D‐POWER are not only locally but also remotely active, thereby enabling a plasmonic web that not only transports plasmonic fields but also carries SERS signals, which can be collected even if the analyte is outside the equipment field of view. In addition, 3D‐POWER is amenable to real‐world applications since it offers scalability, repeatability in terms of sensitivity, cost‐effectiveness (each circular piece of the SERS substrate has an estimated cost of 0.098 USD, see Table S17, Supporting Information), facile fabrication, robust signal intensity and versatility toward the sample incorporation: the SERS substrate can be incubated within liquid samples, or rubbed/adhered into solid surfaces.^[^ ^2^ ^]^ As a proof of concept, we demonstrated that the SERS analysis facilitated by 3D‐POWER is able to reveal the presence of harmful compounds and micro/nanoplastics in food, as well as metabolites in biofluids. This breakthrough in remote SERS might bring radical, yet affordable, analytical applications of ultrasensitive/single molecule Raman spectroscopy and inspire future developments in several fields such as healthcare, nanotoxicology, environmental protection, food safety, nanoscale optical trapping, plasmon‐driven surface‐catalyzed reactions, flexible photonic circuits, hyperbolic metamaterials, and quantum plasmonics.

## 4 Experimental Section

### Reagents

All the employed reagents were of analytical grade and they were handled according to their data sheets. Bacterial Cellulose nanopaper was acquired from Nano Novin Polymer Co. (Mazandaran, Iran). Ultrapure water (resistivity > 18 MΩ cm) was obtained via a Milli‐Q system, Direct‐Q 3UV (Millipore Co., Burlington, MA, USA). Graphene oxide dispersion (N002‐PS‐0.5, GO, 5 mg mL^−1^) was obtained from Angstrom Materials Inc. (Dayton, OH, USA). Silver nitrate (209139, AgNO_3_), Hexadecyltrimetylammonium bromide (H5882, CTAB); Gold (III) chloride trihydrate (520918, HAuCl_4_×3H_2_O), L‐Ascorbic Acid (A7506, AA, C_6_H_8_O_6_) and glyphosate‐2‐^13^C (90007, GLY) were purchased from Sigma‐Aldrich (Toluca, Mexico, Mexico). Fluorescein (free acid), (sc‐215040, FSC) was purchased from Santa Cruz Biotechnology, Inc. (Dallas, Texas, USA); Absolute ethanol (1.07017.4000, EtOH) was purchased by Supelco Merck (Mexico state, Mexico), Sodium borohydride (6029, NaBH_4_) and HPLC grade water (3120, H_2_O) was purchased from Karal, S.A. de C.V. (León, Guanajuato, Mexico).

### Synthesis of 3D‐POWER

*Synthesis of Graphene Oxide‐Decorated Nanopaper*: GO aqueous dispersions were prepared using ultrapure water. Graphene oxide‐decorated nanopaper was synthesized in an Erlenmeyer flask by employing 15 square pieces of BC (2.5×2.5 cm), which were soaked in a 100 mL GO aqueous dispersion (BC/GOXX, 20 ≤ X ≤ 150 µg·mL^−1^ of GO). The mixture was then heated at 90 °C for 2 h with constant stirring. Later GO‐decorated BC was separated from the GO suspension and washed three times with ultrapure water.

*Synthesis of Gold Nanorods (AuNRs)*. A suspension of gold seeds was prepared using 5 mL of HAuCl_4_ concentrated at 0.5 m, which was then mixed with 5 mL of CTAB concentrated at 0.2 M and 0.6 mL of ice‐cold NaBH_4_ concentrated at 0.01 m under vigorous stirring at room temperature. The color of the suspension immediately changed from yellow to brown after NaBH_4_ addition, the suspension was then left to stand for 30 min. A growth suspension was prepared by mixing 100 mL of CTAB concentrated at 0.2 m, 100 mL of HAuCl_4_ concentrated at 1 mm, 3.5 mL of AgNO_3_ concentrated at 4 mM, 1.8 mL of ascorbic acid concentrated at 78 mm, and 450 µL of the freshly prepared seed suspension was added to the growth suspension. The mixture was then left to stand for 3 h at room temperature. Finally, the AuNRs suspension was centrifuged for 1.5 h, at 4500 rpm. The AuNRs were then separated and dispersed in 100 mL of ultrapure water.

*Synthesis of BC/GO/AuNRs*. Ten pieces of BC/GO (nanopapers previously decorated with GO) were mixed in a 100 mL suspension of AuNRs concentrated at 0.7 mm by gentle stirring for 12 h (generally 20 mL of the previously prepared AuNRs were diluted in ultrapure water using a final volume of 100 mL). Eventually, the biohybrid material (BC/GO/AuNRs) was separated and washed three times with ultrapure water and two times with water/ethanol (50% v/v) dried and stored at room temperature in dark conditions. Generally, the synthesized substrates were cut in millimetric circles (diameter of 6 mm) using punch tools before usage.

### Materials Characterization

UV–vis absorption spectroscopy was used to monitor the extinction (absorbance, reflection, and scattering) of the studied materials. UV–vis spectra were recorded using a spectrophotometer Cytation 5, BioTek (Winooski, Vermont, USA).

High‐resolution field emission scanning electron microscopy was performed using a JEOL‐JSM‐7800F equipment (Akishima, Tokio, Japan); acceleration voltage of 1 kV and a working distance of 4 mm.

### Raman Analysis

Raman and SERS spectra were acquired using a Renishaw inVia Raman module for spectroscopic analysis (Wotton‐under‐Edge, UK), which was coupled on a Leica DM 2500M microscope in vertical configuration. A 50X objective (numerical aperture, 0.75) was employed in all the experiments. The red excitation line was provided by a diode laser centered at 785 nm (0.08 mW), polarization state R. Importantly, a series of in silico experiments confirmed that the employed laser line (785 nm) is optimal to produce and study the innovative plasmonic web discussed in the research, see the Video S1 (Supporting Information). Video S1 (Supporting Information) depicts a 3D representation of the plasmonic web pumped throughout the visible spectra (1000 nm to 450 nm) and generating along this in silico experiment localized plasmons far from the excitation source, especially with more intensity at c.a. 780 nm. All the SERS substrates were analyzed once they were completely dried, after analyte incubation.

### Statistical Analysis and Raman Spectra Treatment

The recorded spectra were background/baseline subtracted from 600 to 1700 cm^−1^ in MATLAB R2023a Software. The SERS/Raman signals were normalized according to the most intense vibrational band, respectively. Generally, each spectrum represented the average of at least one hundred spectra recorded onto an area of 2500 µm^2^ of 3D‐POWER. The error bars depicted in the graphs represent the respective standard deviation.

### Determination of the Analytical Enhancement Factor

Raman signals were obtained by incubating, overnight, circular substrates of BC (diameter, 6 mm) in 1 mL of FSC or GLY concentrated at 1×10^−3^ m, respectively. The same experimental conditions were employed: excitation wavelength of 785 nm; power, 0.08 mW; size of the spot 1.2 µm, number of acquisitions, 10 and exposure time, 2 s; see Figure S15 (Supporting Information). The analytical enhancement factor was estimated by determining the relative ratio SERS signal / Raman signal, particularly using Equation (6) reported by Le Ru and Etchegoin.^[^ ^53^ ^]^

(1) $$\mathrm{AEF}=\left(I_{\mathrm{SERS}}/C_{\mathrm{SERS}}\right)/\left(I_{\mathrm{Raman}}/C_{\mathrm{Raman}}\right)$$

where I_Raman_ is the Raman intensity obtained at a specific Raman shift at the analyte concentration C_Raman_ (in the absence of the SERS substrate). I_SERS_ is the SERS intensity obtained at a specific Raman shift with the SERS substrate at the analyte concentration C_SERS_, using the same experimental conditions in the Raman signal acquisition process. In the determination of the analytical enhancement factor for FSC analysis, the employed Raman shift was 1616 cm^−1^ and C_Raman_ = 1×10^−3^ m, I_Raman_ = 879.98, see Table S9 (Supporting Information). In the determination of the analytical enhancement factor for GLY analysis, the employed Raman shift was 1500 cm^−1^ and C_Raman_ = 1.8×10^−3^ m, I_Raman_ = 140.64, see Table S12 (Supporting Information).

### Analysis of Liquid Samples

SERS substrates were incubated overnight, at room temperature, in 1 mL of liquid samples containing the analytes such as Fluorescein (FSC) and Glyphosate (GLY) (both analytical standard grade) at different concentrations (ex. mm ≤ [analyte] ≤ ym), which were prepared by serial dilutions. FSC was diluted using absolute ethanol. GLY was diluted using HPLC grade water.

### Corn analysis

Sample A of corn was a commercially available cornmeal of white corn. Sample B of corn was a commercially available cornmeal of blue corn. Sample C was prepared by milling of the kernels of a white corn grown in the southern region of Mexico. In all cases, 10 milligrams of cornmeal for each sample was diluted in HPLC grade water in the range of milligrams to yoctograms, which were prepared using serial dilutions. Finally, one SERS substrate (diameter of 6 mm) was incubated overnight in each liquid sample, just as shown in the insert of Figure 4a. The respective Raman mapping was obtained through an excitation wavelength of 785 nm; power, 0.08 mW; size of the spot 1.2 µm number of acquisitions, 10 and exposure time, 2 s.

### Vegetables and Fruits Analysis

The SERS substrates were rubbed for ten seconds onto the surface of an apple, orange, tomato, mango, and lettuce, respectively. The respective Raman mapping was obtained through an excitation wavelength of 785 nm; power, 0.08 mW; size of the spot 1.2 µm; number of acquisitions, 10 and exposure time, 2 s.

### Sweat Analysis

Four male volunteers participated in this section of the research. All the volunteers run for 15 min to promote sweat excretion. The SERS substrate was placed onto the temple of the volunteer, and the substrate was impregnated with sweat for 5 min. All volunteers expressed the informed consent. The respective Raman mapping was obtained through an excitation wavelength of 785 nm; power, 0.8 mW; size of the spot 1.2 µm; number of acquisitions, 10 and exposure time, 2 s.

### Sebum Analysis

One male volunteer and one female volunteer participated in this section of the research. The SERS substrate was rubbed onto the “T zone” of the face of the volunteers for 5 s. The volunteers did not employ any cosmetic product in the face before sebum sample acquisition. The respective Raman mapping was obtained through an excitation wavelength of 785 nm; power, 0.8 mW; size of the spot 1.2 µm; number of acquisitions, 10 and exposure time, 2 s.

### In Silico Experiments

Simulations were performed by using the Electromagnetic Waves module and Frequency Domain study offered by the software COMSOL‐Multyphysics 5.2a. A working wavelength of 785 nm and an incident plane wavefront were considered in this series of experiments (the same as those conditions employed in the SERS experiments). Table S13 (Supporting Information) shows the properties (permittivity, permeability, and conductivity) of the materials that were employed to perform the simulations. Scattering boundary conditions were respectively defined for the field emission and for the perfect conductor along the AuNRs boundaries. Each GO film, with 1 nm of thickness, represents a complex dielectric material in the visible and near‐infrared wavelength range that allows light confinement and propagation.^[^ ^54^ ^]^ AuNRs with 50 nm x 14 nm were considered in this series of in silico experiments. Given the scale of the simulated composite material, the selected scale was in nanometers. Considering these parameters, two types of in silico experiments were performed: simulations representing the electromagnetic enhancement offered by the studied materials, such as those depicted in Figure 1f,j and Figures S3–S6 (Supporting Information); and simulations representing the plasmonic transport offered by the studied materials, such as those depicted in Figure 3a and Figures S16 and S17 (Supporting Information). The workflow followed in these simulations is depicted in Scheme S3 (Supporting Information). The employed meshing is depicted in Scheme S4 (Supporting Information).

Electromagnetic enhancement simulations started in a plane, where the AuNRs have ellipsoidal geometries, and GO monolayers as well as BC represented the involved media. A triangular mesh, in an extra fine configuration was employed, which ranged from 0.1 to 2 nm. To generate an incident plane wave into the media, in the electromagnetic enhancement simulations, the boundaries were specifically defined as a source of light, while all the AuNRs boundaries were defined as perfect electric conductors.

In the light propagation simulations, the AuNRs were designed from the Boolean intersection between spheres and cylinders. GO and BC were defined by blocks, representing the surrounding media. The mesh was composed of tetrahedrons in an extra fine configuration that ranged from 0.1 to 2 nm. A truncated cone was used as a source of the incident electric field. Hence, the scattering boundary conditions are set‐up by the semi‐plane cone (the electric field source), and the AuNRs (perfect electric conductors).

## Conflict of Interest

The authors declare no conflict of interest.

## Supporting information

Supporting Information

Supporting Information 1

## Data Availability

The data that support the findings of this study are available in the supplementary material of this article.
