# Correction to “Flexible 3D Plasmonic Web Enables Remote Surface Enhanced Raman Spectroscopy”

**DOI:** 10.1002/advs.202412492

**Published:** 2024-11-04

**Authors:** E. Rodríguez‐Sevilla, J. U. Álvarez‐Martínez, R. Castro‐Beltrán, E. Morales‐Narváez

*Adv. Sci*. **2024**, *11*, 2402192.

https://doi.org/10.1002/advs.202402192

We committed a series of errors that led us to assume that glyphosate (GLY, CAS 287399‐31‐9), one of the model analytes, was analyzed at the yM level as follows: First, we had an error related to units’ conversion, hence mathematically (but not experimentally) our analysis was virtually carried out in a volume of 100 L (instead of 1 mL, which was the experimental volume). Consequently, we wrongly assumed that those spectra related to GLY concentrated at ym level and lower concentration levels were feasible to record. However, as discussed above, the analysis of GLY at ym level (and lower concentration levels) is only achievable in the analysis of a volume of 100 L. Hence, we recognize that those spectra originally attributed to GLY concentrated at ym level and lower concentration levels correspond to contaminated samples. Consequently, a series of corrections are detailed below. We also recognize that given the ultrasensitive character of the reported innovative surface‐enhanced Raman spectroscopy (SERS) substrate, the substrate can be easily contaminated.

This series of errors does not affect our findings and conclusions since the ultrasensitive character and the operating mechanism of our innovative SERS substrate is supported by the overall content of the manuscript. The authors regret this scenario and apologize for any inconvenience caused.

**Detailed list of corrections**:

**Text**

**Table:** 

Section	Incorrect term	Correct term
Abstract (page 1, paragraph 1, line 17)	yoctomolar	zeptomolar
Introduction (page 2, paragraph 3, line 5)	yoctomolar	zeptomolar
Results and Discussion / 2.2 Surface Enhanced Raman Spectroscopy Behavior (page 4, paragraph 2, line 18)	yoctomolar	zeptomolar
Results and Discussion / 2.2 Surface Enhanced Raman Spectroscopy Behavior (the Analytical Enhancement Factor, page 5, Paragraph 3, line 21)	6 × 10^21^	1.5 × 10^18^
Experimental section / Corn analysis (page 10, line 6).	yoctograms	zeptograms
Experimental section / Analysis of liquid samples (page 10, line 4)	ym	zM
Figure 2 caption.	yM	zm
Figure 2 caption	[GLY] = 10^−24^ m	[GLY] = 10^−21^ m
Table 1, row 4	(AEF) 6 × 10^21^	(AEF) 1.5 × 10^18^

**Figures**

Figure 2 (panel c,d) needs a correction which is reflected in the updated figure below. Particularly, in Figure 2c, the curves related to yM and 10^−27^ M were removed in the corrected Figure 2. In addition, the corrected Figure 2d displays the SERS spectra corresponding to the analysis of GLY concentrated at 10^−21^ M.

**Figure 2:**
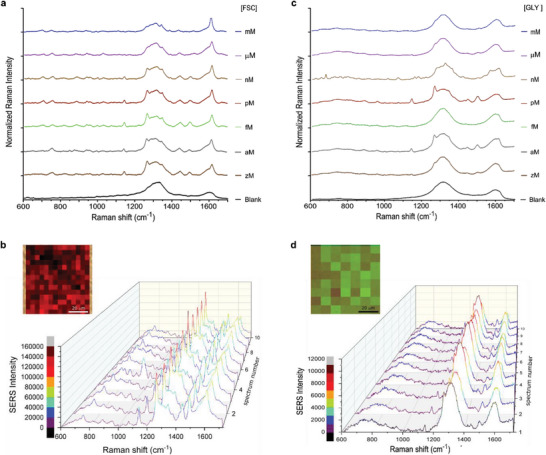
3D‐POWER as a SERS substrate. a). SERS spectra of 3D‐POWER impregnated with FITC (FSC) at different concentrations (from mM to zm). b). Representative SERS spectra and SERS mapping (relative intensity of the peak at 1616 cm^−1^) offered by 3D‐POWER impregnated with [FSC] = 10^−21^ m (zM). c). SERS spectra of 3D‐POWER impregnated with Glyphosate (GLY) at different concentrations (from mm to zM). d). Representative SERS spectra and SERS mapping (relative intensity of the peak at 1500 cm^−1^) offered by 3D‐POWER impregnated with [GLY] = 10^−21^ m (zm). Each spectrum represents the average of at least one hundred spectra recorded onto an area of 2500 µm^2^ of 3D‐POWER. All Raman measurements were performed using an excitation wavelength 785 nm, power, 0.08 mW, and exposure time, 2 s, size of the spot, 1.2 µm, and number of acquisitions, 10.

**Supporting Information**

Figure S15 (page 19 of the Supporting Information) needs a correction according to the updated Figure below


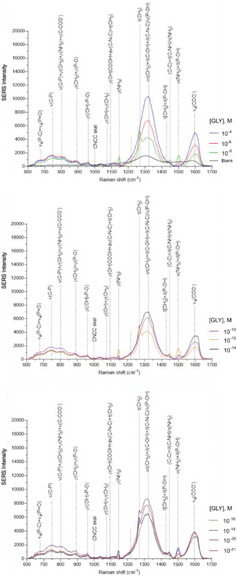


Figure S15. Detailed SERS spectra of GLY analyzed at different concentrations using 3D‐POWER (BC/GO35/AuNRs). Each spectrum represents the mean of fifty spectra recorded on 2500 µm^2^ of the corresponding SERS substrate. The spectra were obtained through: excitation wavelength, 785 nm; laser power, 0.08 mW; size of the spot, 1.2 µm; exposure time, 2 s and number of acquisitions, 10.

Table S10 (page 36 of the Supporting Information) needs a correction according to the updated table below

Table S10. Estimation of the number of GLY molecules.

**Table:** 

Experimental concentration (mol L^−1^)	Moles number	Molecules number
5.88E‐03	5.88E‐06	4E + 18
5.88E‐04	5.88E‐07	4E + 17
5.88E‐05	5.88E‐08	4E + 16
5.88E‐06	5.88E‐09	4E + 15
5.88E‐07	5.88E‐10	4E + 14
5.88E‐08	5.88E‐11	4E + 13
5.88E‐09	5.88E‐12	4E + 12
5.88E‐10	5.88E‐13	4E + 11
5.88E‐11	5.88E‐14	4E + 10
5.88E‐12	5.88E‐15	4E + 09
5.88E‐13	5.88E‐16	4E + 08
5.88E‐14	5.88E‐17	4E + 07
5.88E‐15	5.88E‐18	4E + 06
5.88E‐16	5.88E‐19	4E + 05
5.88E‐17	5.88E‐20	4E + 04
5.88E‐18	5.88E‐21	4E + 03
5.88E‐19	5.88E‐22	354.09
5.88E‐20	5.88E‐23	35.41
5.88E‐21	5.88E‐24	3.54

SERS substrate: BC/AuNRs or BC/GO35/AuNRs, BC, bacterial nanocellulose; GO, graphene oxide; AuNRs, gold nanorods, [GLY], concentration of glyphosate, M, mol L^−1^.

Table S12 (page 38 of the Supporting Information) needs a correction according to the updated table below

Table S12. Analytical enhancement factor for GLY via 3D‐POWER.

**Table:** 

[GLY], [M]	Intensity at Raman shift				Molecules number	AEF
	880 cm^−1^	970 cm^−1^	1417 cm^−1^	1500 cm^−1^		
5.88E‐04	811	315	1114	150	4E + 17	3.3E + 00
5.88E‐06	860	316	697	527	4E + 15	1.1E + 03
5.88E‐08	796	347	571	1641	4E + 13	3.6E + 05
5.88E‐10	937	359	671	142	4E + 11	3.1E + 06
5.88E‐12	820	379	418	1669	4E + 09	3.6E + 09
5.88E‐14	754	266	633	579	4E + 07	1.3E + 11
5.88E‐16	662	277	734	130	4E + 05	2.8E + 12
5.88E‐18	733	236	676	1573	4E + 03	3.4E + 15
5.88E‐20	1159	440	908	1272	35.41	2.8E + 17
5.88E‐21	744	258	856	683	3.54	1.5E + 18

BC, bacterial nanocellulose; GO35, graphene oxide at 35 µg·mL^−1^; AuNRs, gold nanorods;[GLY], concentration of glyphosate, M, mol L^−1^. The AEF, Analytical Enhancement Factor, was obtained through the Raman intensity recorded in BC, GLY was concentrated at 1.8x10^−3^ M, the Raman intensity at 1500 cm^−1^ was employed to this end (see the Experimental Section).

## Supporting information



Supporting Information

