# Supplementary material for: Flexible 3D Plasmonic Web Enables Remote Surface Enhanced Raman Spectroscopy
Source: Adv Sci (Weinh). 2024 Apr 6;11(23):2402192. doi: 10.1002/advs.202402192 (PMC11187956; doi:10.1002/advs.202402192)
Supplement: Supplementary file 1 — Supporting Information [file ADVS-11-2402192-s001.pdf]

ADVANCED SCIENCE
Open Access

Supporting Information

for *Adv. Sci.*, DOI 10.1002/advs.202402192

Flexible 3D Plasmonic Web Enables Remote Surface Enhanced Raman Spectroscopy

*Erika Rodríguez-Sevilla, Jonathan Ulises Álvarez-Martínez, Rigoberto Castro-Beltrán and Eden Morales-Narváez**

Supporting Information

Flexible 3D Plasmonic Web Enables Remote Surface Enhanced Raman Spectroscopy

*Erika Rodríguez-Sevilla, Jonathan Ulises Álvarez-Martínez, Rigoberto Castro-Beltrán, Eden Morales-Narváez**

Table of content

Scheme S1. Fabrication of BC/GO substrate. **i**. BC is washed with ultrapure water. ii. BC is added in a GO suspension. GO is spontaneously self-assembled by layers within BC. **iii**. BC/GO is washed and dried. ____ **4**

Scheme S2. Gold nanorods (AuNRs) synthesis (by growth seed method). ____ **4**

Figure S1. Raman spectra recorded on bacterial nanocellulose (BC) as a substrate. ____ **5**

Figure S2. SEM micrographs of different configurations of the explored biohybrid: superficial view.. **6**

Figure S3. SEM micrographs of different configurations of the explored biohybrid: transversal view.. **7**

Figure S4. *In silico* experiments demonstrating the electromagnetic field distribution in BC/GO35/AuNRs, horizontal plane. ____ **8**

Figure S5. *In silico* experiments demonstrating the electromagnetic field distribution in BC/GO35/AuNRs, frontal plane. ____ **9**

Figure S6. *In silico* experiments demonstrating the electromagnetic field distribution in BC/AuNRs, horizontal plane. ____ **10**

Figure S7. *In silico* experiments demonstrating the electromagnetic field distribution in BC/AuNRs, frontal plane. ____ **11**

Figure S8. UV-Vis characterization of the synthesized materials. ____ **12**

Figure S9. a. Species predominance diagram of FSC. **b.** SERS spectra facilitated by 3D-POWER. ____ **13**

Figure S10. SERS analysis of FSC at several concentrations. ____ **14**

Figure S11. Analysis of repeatability and signal intensity robustness. (**a-c**) SERS spectra of FSC at different concentrations, obtained with 3D-POWER (BC/GO35/AuNRs) fabricated in different batches. ____ **15**

Figure S12. SERS spectra of FSC at several concentrations analyzed with different SERS substrates. **16**

Figure S13. Detailed SERS spectra of FSC analyzed at different concentrations using 3D-POWER ____ **17**

Figure S14. a. Species predominance diagram of GLY, **b.** SERS spectra facilitated by 3D-POWER. ____ **18**

Figure S15. Detailed SERS spectra of GLY analyzed at different concentrations using 3D-POWER (BC/GO35/AuNRs). ____ **19**

Figure S16. In silico experiments were performed to investigate the light transport capability of the materials composing 3D-POWER. 20

Figure S17. 3D simulations (x = 800 nm, y = 800 nm and z = 50 nm) revealed that 3D-POWER was able to capture light in a small region (5 x 10^5 nm^3) and perform plasmonic field transmission, thereby generating a plasmonic web throughout the simulated volume. 21

Figure S18. Dark field images of 3D-POWER demonstrating bright emissions from plasmonic nanoparticles beyond the laser spot. 22

Figure S19. Remote SERS experiment using a rectangular piece of 3D-POWER. 23

Figure S20. a. 0.1 µL of FSC concentrated at 10^{-16} M (c.a. 10 molecules) were drop casted onto the center of a circular piece of 3D-POWER. 24

Figure S21. Corn analysis. 25

Figure S22. Pesticide detection using 3D-POWER as SERS substrate in commercially available cornmeal (white corn: Sample A; blue corn: Sample B) and non-commercially available white cornmeal (Sample C). 26

Figure S23. Food analysis. 27

Scheme S3. Flowchart followed in the *in silico* experiments. 28

Scheme S4. a. Graphical representation of the meshing employed in simulations representing the electromagnetic enhancement offered by the studied materials. 29

Table S1. (Nano)metrology and physicochemical features of each component of 3D-POWER 30

Table S2. UV-Vis features of the explored SERS substrates. 30

Table S3. Particle density in the studied biohybrids. 31

Table S4 Mean values and ranges of the resulting electromagnetic enhancement factors (γ) in the explored materials. 31

Table S5 Estimation of the number of FSC molecules. 31

Table S6. Statistical analysis of the explored SERS substrates. FSC was employed as a model analyte. 32

Table S7. Assessment of the behavior of BC/GO35/AuNRs in different batches. FSC was employed as a model analyte. 33

Table S8. Vibrational bands observed in the SERS spectra of FSC (diluted in ethanol). 34

Table S9. Analytical enhancement factor observed in FSC via 3D-POWER. 35

Table S10. Estimation of the number of GLY molecules. 36

Table S11. Vibrational bands observed in in the analysis of GLY concentrated at 10^{-9} M (diluted in HPLC grade water). 37

Table S12. Analytical enhancement factor for GLY via 3D-POWER. 38

Table S13. Material properties considered in the *in silico* experiments. 38

Table S14. Vibrational bands observed using 3D-POWER, which were rubbed into the surface of vegetables and fruits. 39

Table S15. Vibrational bands observed using 3D-POWER substrates. ___ **40**

Table S16. Vibrational bands observed using 3D-POWER substrates. ___ **40**

Table S17. Cost estimation to 3D-POWER fabrication___ **41**

Selection of the optimal SERS substrate. ___ **41**

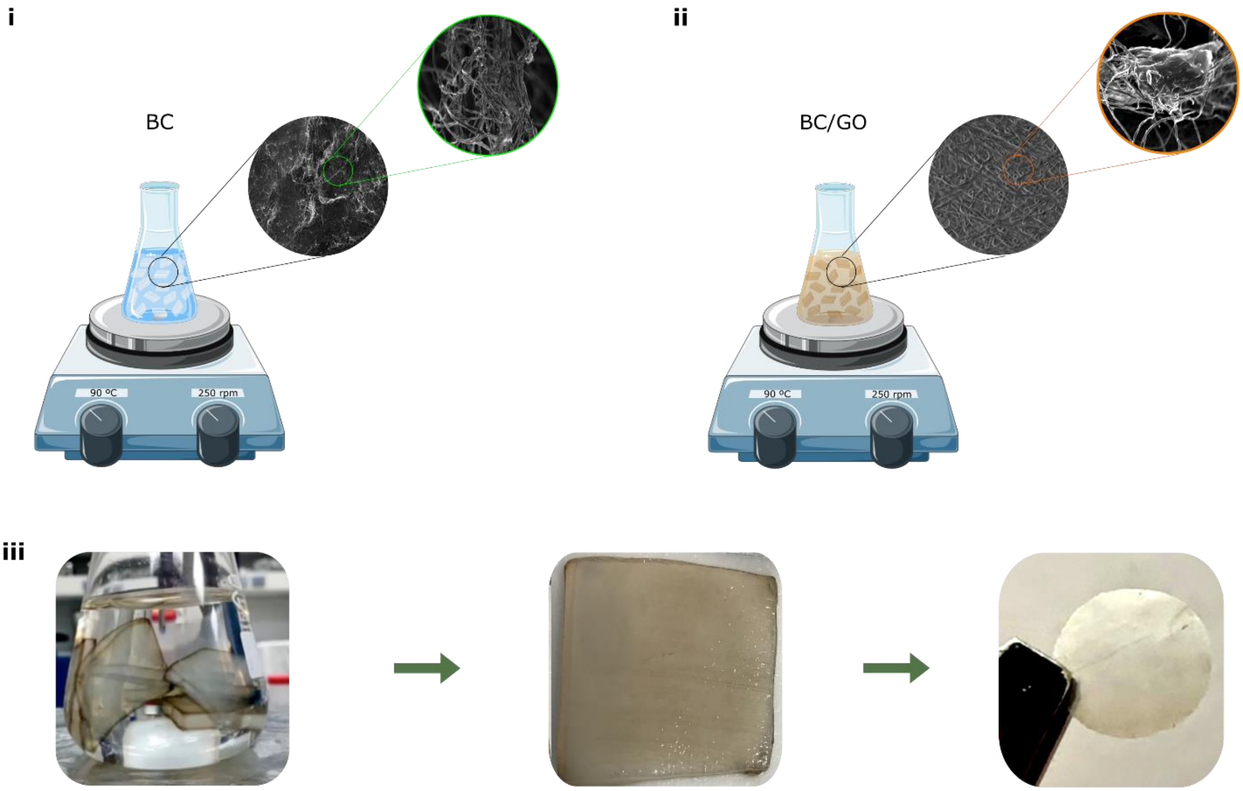

Scheme S1. Fabrication of BC/GO substrate. **i**. BC is washed with ultrapure water. ii. BC is added in a GO suspension. GO is spontaneously self-assembled by layers within BC. **iii**. BC/GO is washed and dried.

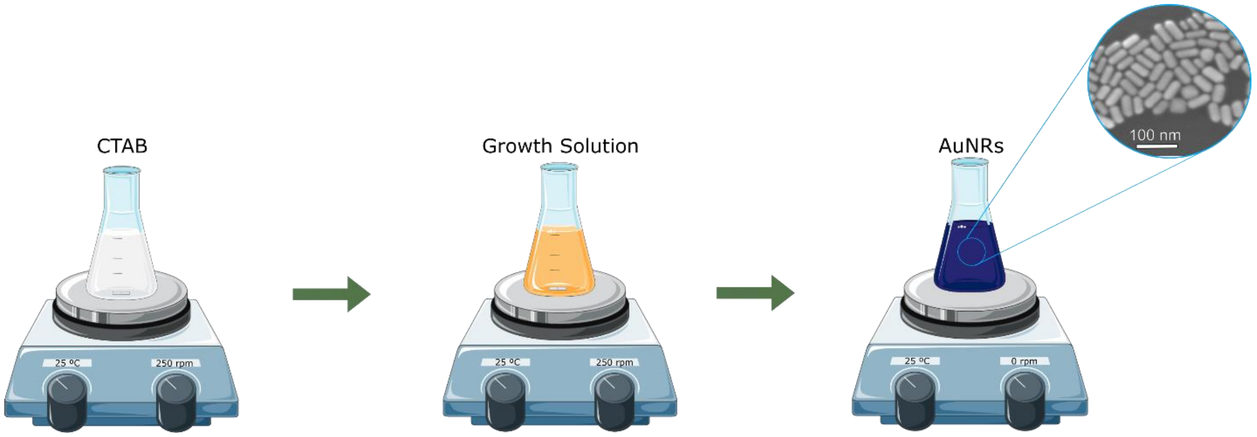

Scheme S2. Gold nanorods (AuNRs) synthesis (by growth seed method).

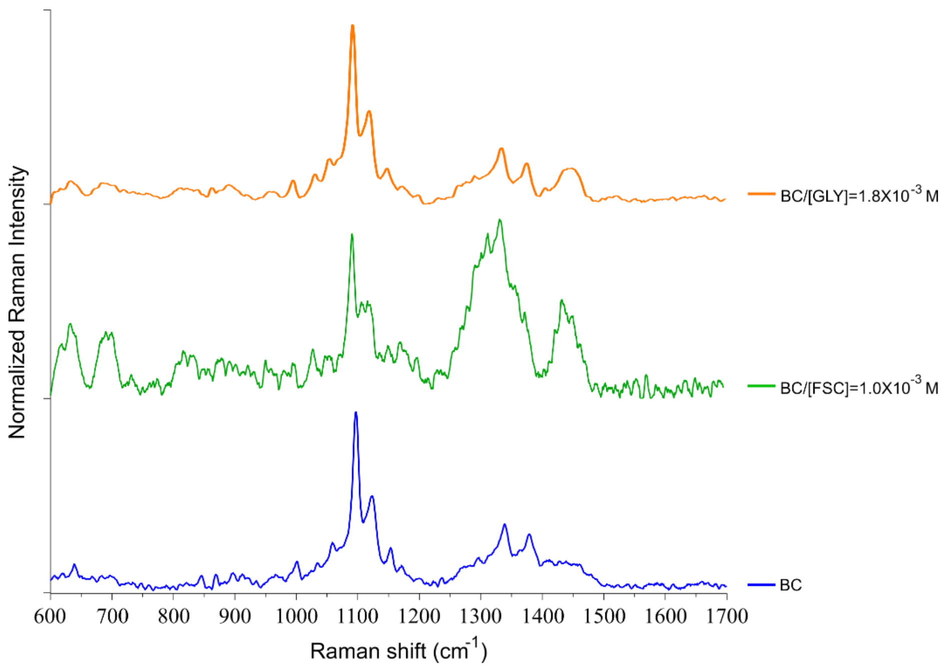

Figure S1. Raman spectra recorded on bacterial nanocellulose (BC) as a substrate. The substrate (BC) was incubated overnight in 1 mL to Fluorescein (FSC) or Glyphosate (GLY) concentrated at $1.0X10^{-3}$ M and $1.8X10^{-3}$ M respectively. Each spectrum represents the mean of fifty spectra recorded on 2500 μm^2 of the corresponding substrate. Excitation wavelength, 785 nm; laser power 0.08 mW; size of the spot = 1.2 µm; exposure time, 2 s. number of acquisitions, 10.

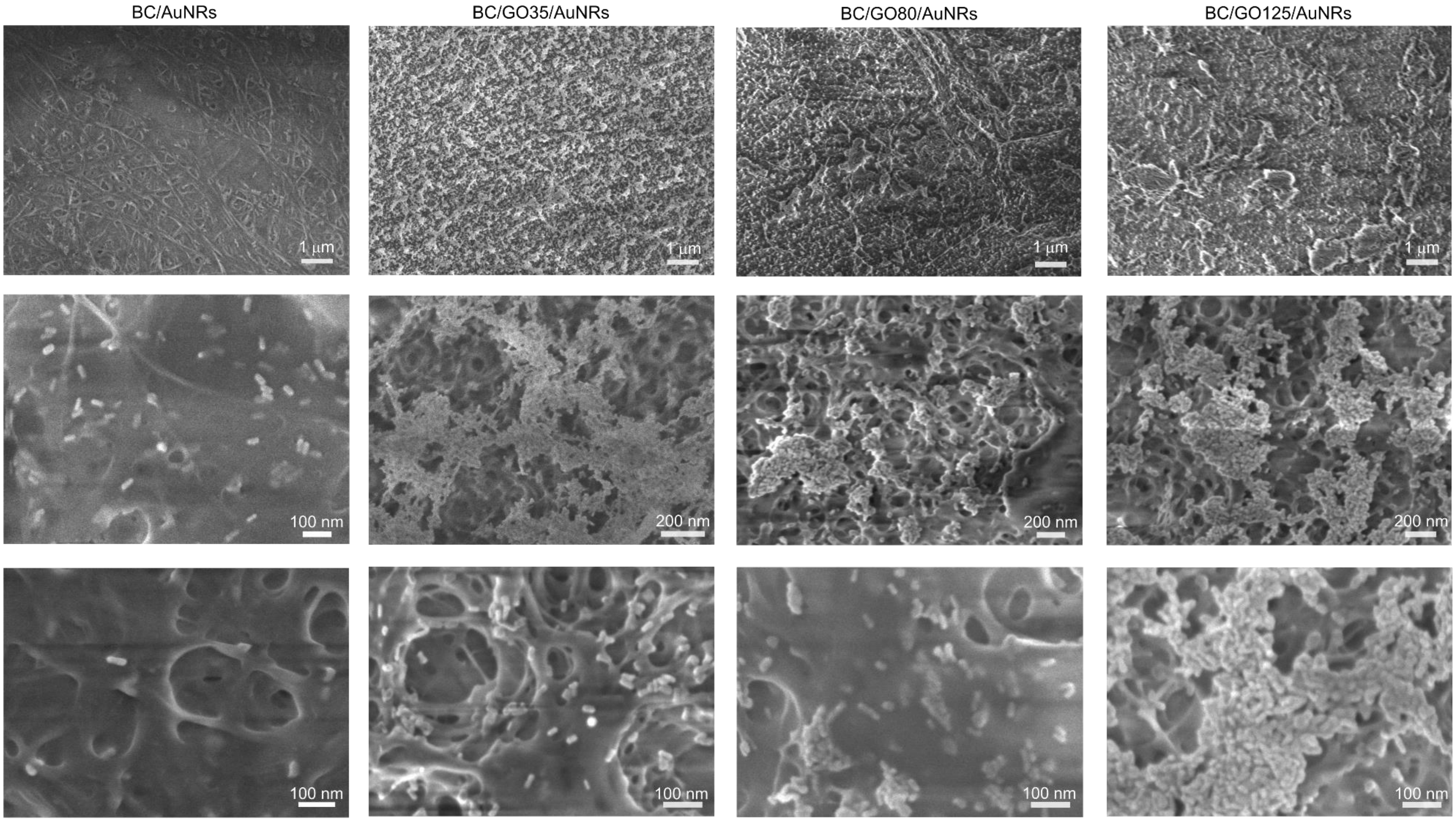

Figure S2. SEM micrographs of different configurations of the explored biohybrid: superficial view. First column, BC/AuNRs; second column BC/AuNRs fabricated in the presence of 35 µg mL-1 of GO; third column BC/AuNRs fabricated in the presence of 80 µg mL-1 of GO and forth column BC/AuNRs fabricated in the presence of 125 µg mL-1 of GO.

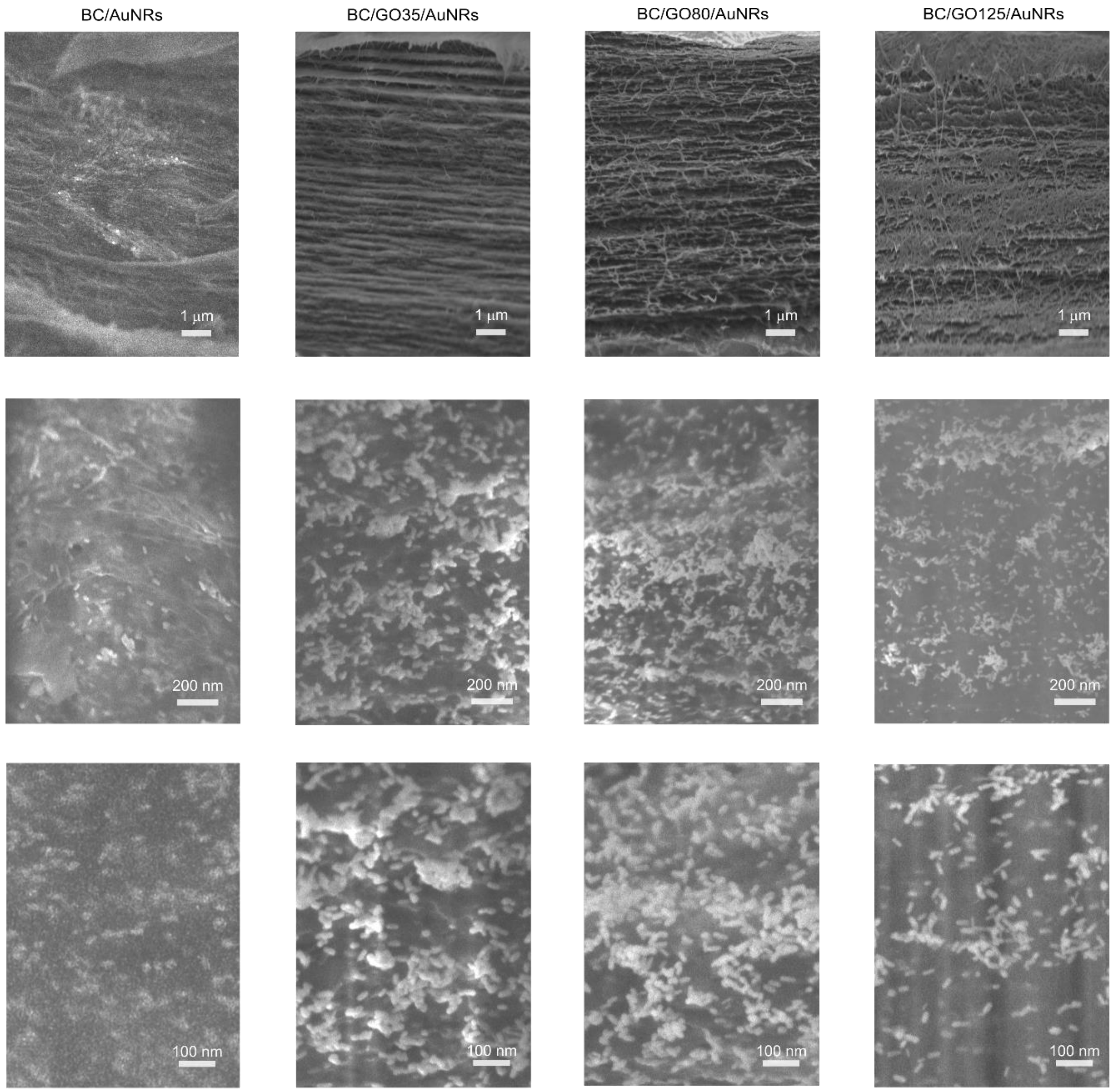

Figure S3. SEM micrographs of different configurations of the explored biohybrid: transversal view. First column, BC/AuNRs; second column BC/GO35/AuNRs (fabricated in the presence of 35 µg mL^{-1} of GO); third column BC/GO80/AuNRs (fabricated in the presence of 80 µg mL^{-1} of GO) and fourth column BC/GO125/AuNRs (fabricated in the presence of 125 µg mL^{-1} of GO).

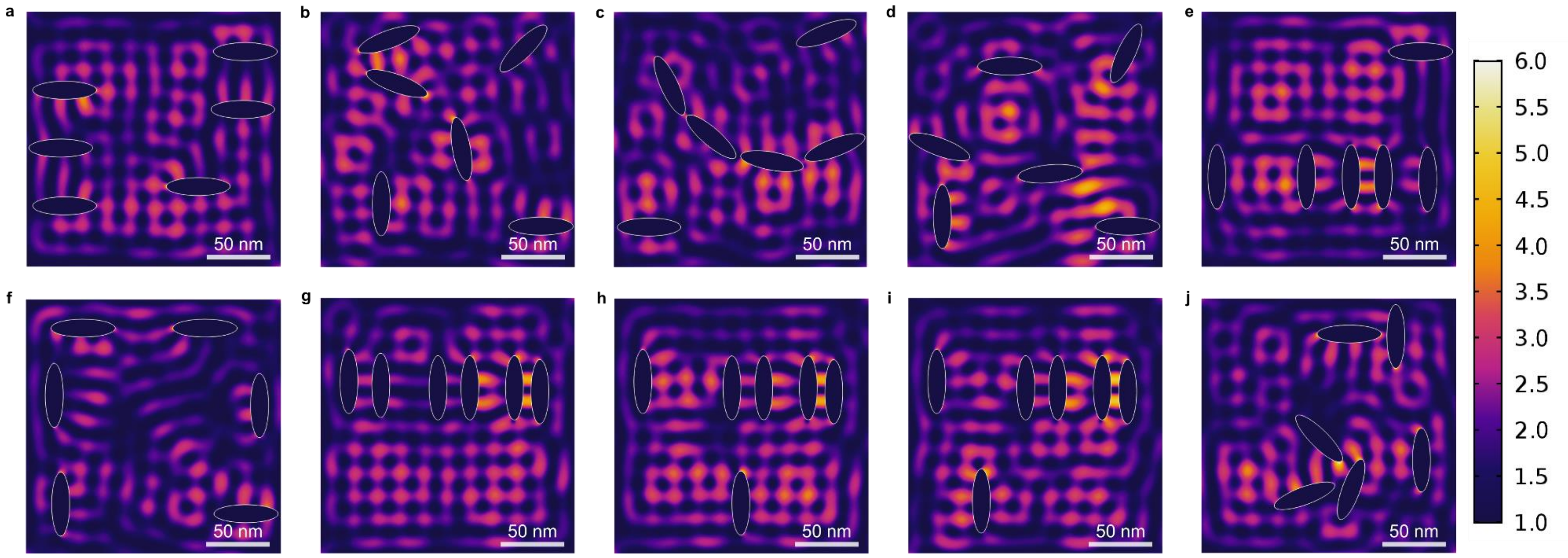

Figure S4. *In silico* experiments demonstrating the electromagnetic field distribution in BC/GO35/AuNRs, horizontal plane. The panels display 10 different scenarios, where, considering the respective particle density (see Table S3), the AuNRs were randomly oriented.

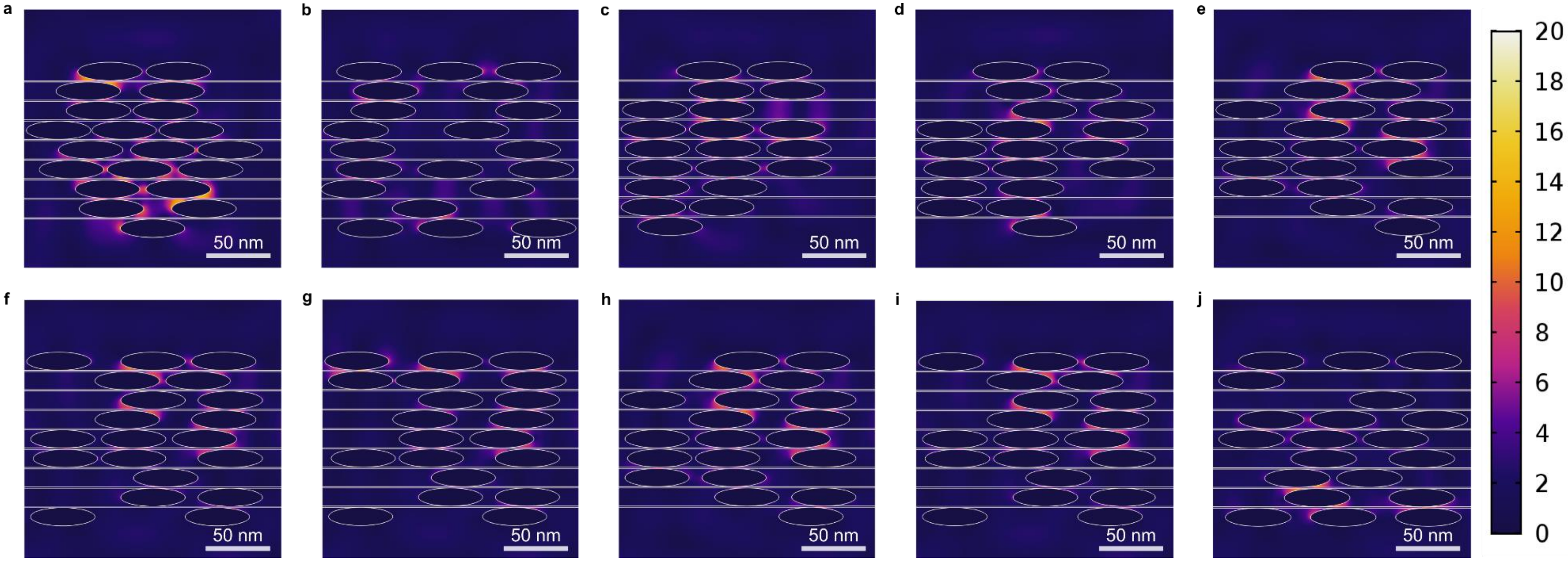

Figure S5. *In silico* experiments demonstrating the electromagnetic field distribution in BC/GO35/AuNRs, frontal plane. The panels display 10 different scenarios, where, considering the respective particle density (see Table S3), the AuNRs were randomly oriented.

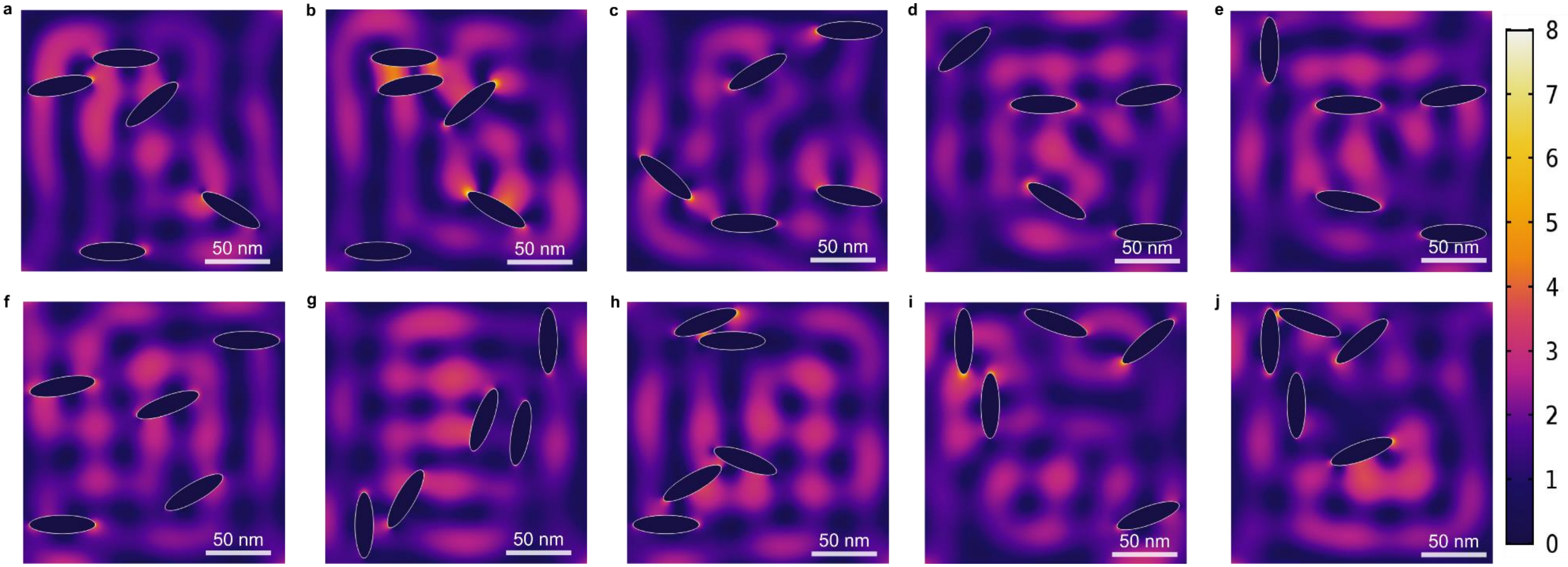

Figure S6. *In silico* experiments demonstrating the electromagnetic field distribution in BC/AuNRs, horizontal plane. The panels display 10 different scenarios, where, considering the respective particle density (see Table S3), the AuNRs were randomly oriented.

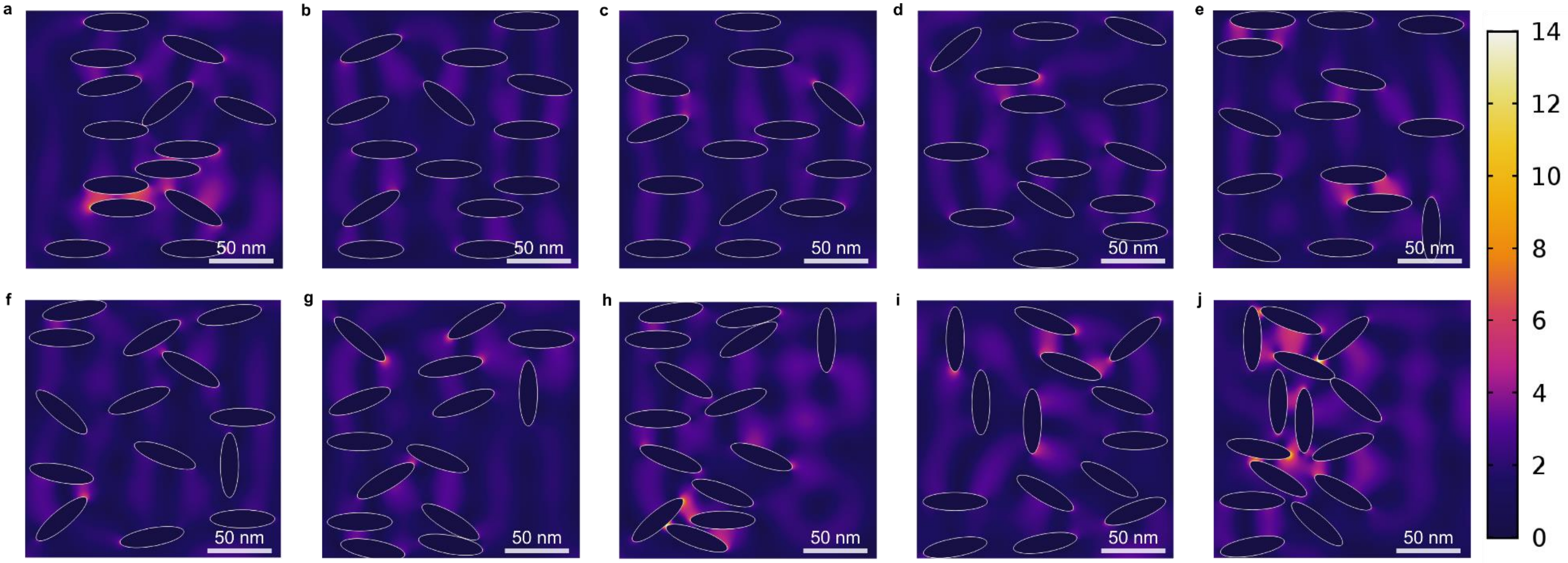

Figure S7. *In silico* experiments demonstrating the electromagnetic field distribution in BC/AuNRs, frontal plane. The panels display 10 different scenarios, where, considering the respective particle density (see Table S3), the AuNRs were randomly oriented.

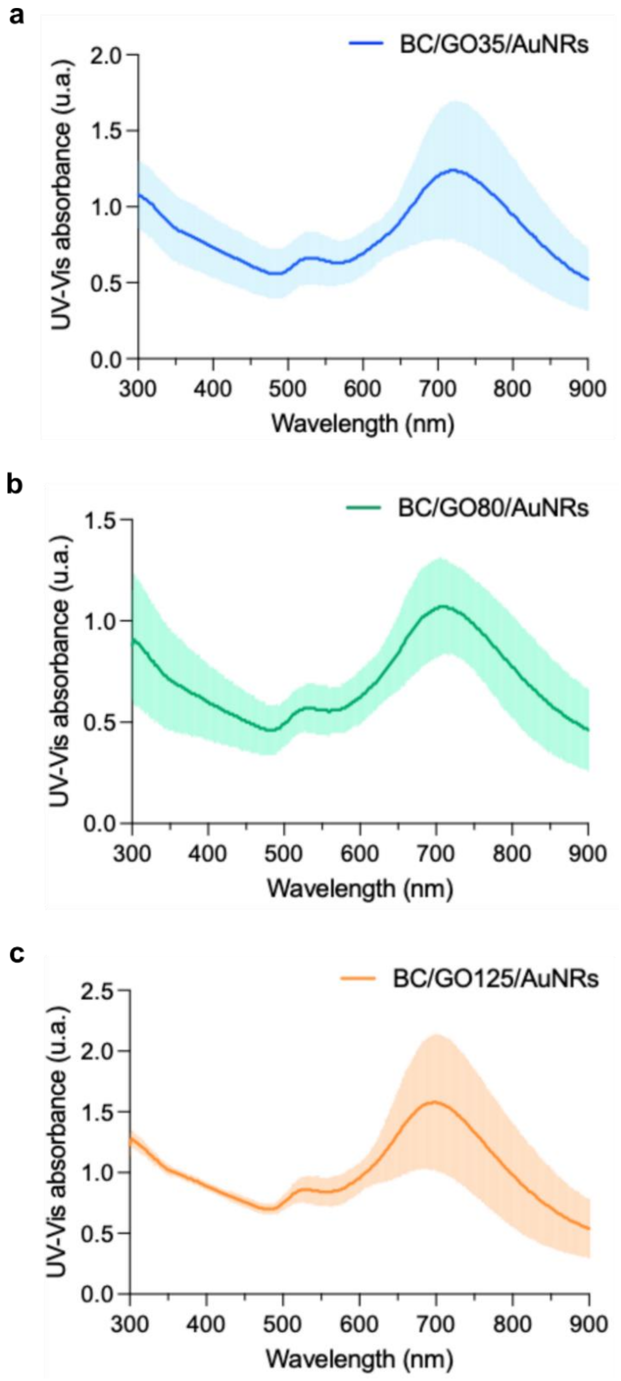

Figure S8. UV-Vis characterization of the synthesized materials. Mean UV-Vis absorption spectra, obtained by averaging the spectra of 15 samples (three samples per batch, five different batches). **a.** BC/GO35/AuNRs, **b.** BC/GO80/AuNRs, **c.** BC/GO125/AuNRs. The shaded area represents the standard deviation (*n* = 15).

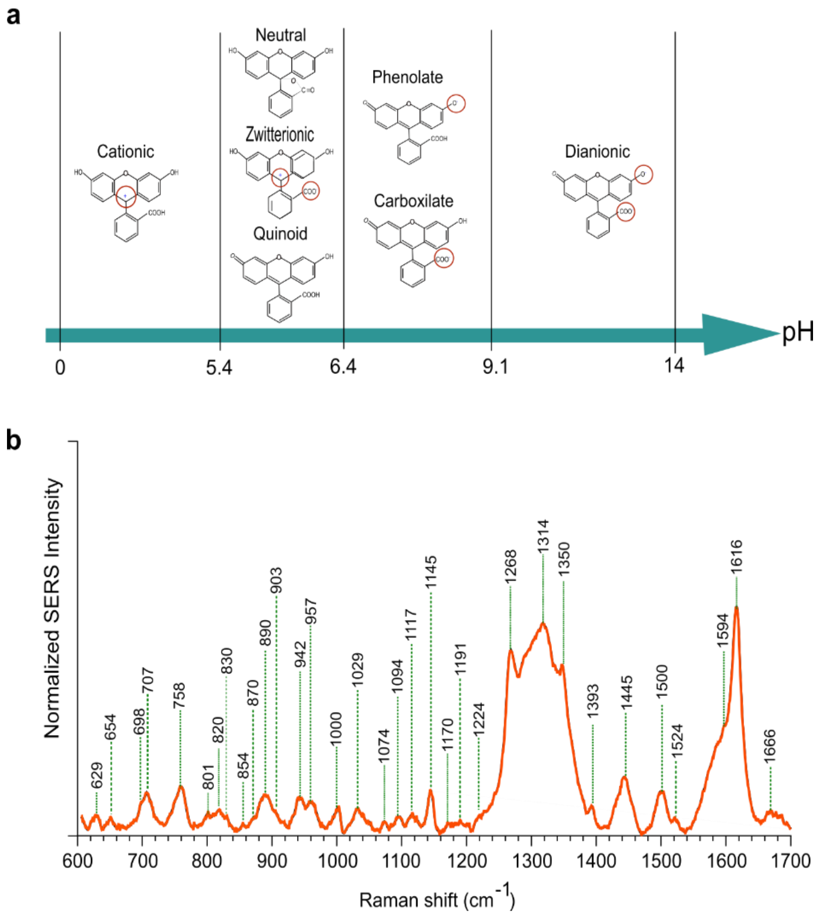

Figure S9. a. Species predominance diagram of FSC. **b.** SERS spectra facilitated by 3D-POWER. The SERS substrate (BC/GO35/AuNRs) was incubated overnight in 1 mL of FSC concentrated at 10^{-9} M. The spectrum represent the mean of fifty spectra recorded on 2500 μm^2 of the corresponding SERS substrate, obtained through: excitation wavelength, 785 nm; laser power 0.08 mW; size of the spot = 1.2 µm; exposure time, 2 s; number of acquisitions, 10.

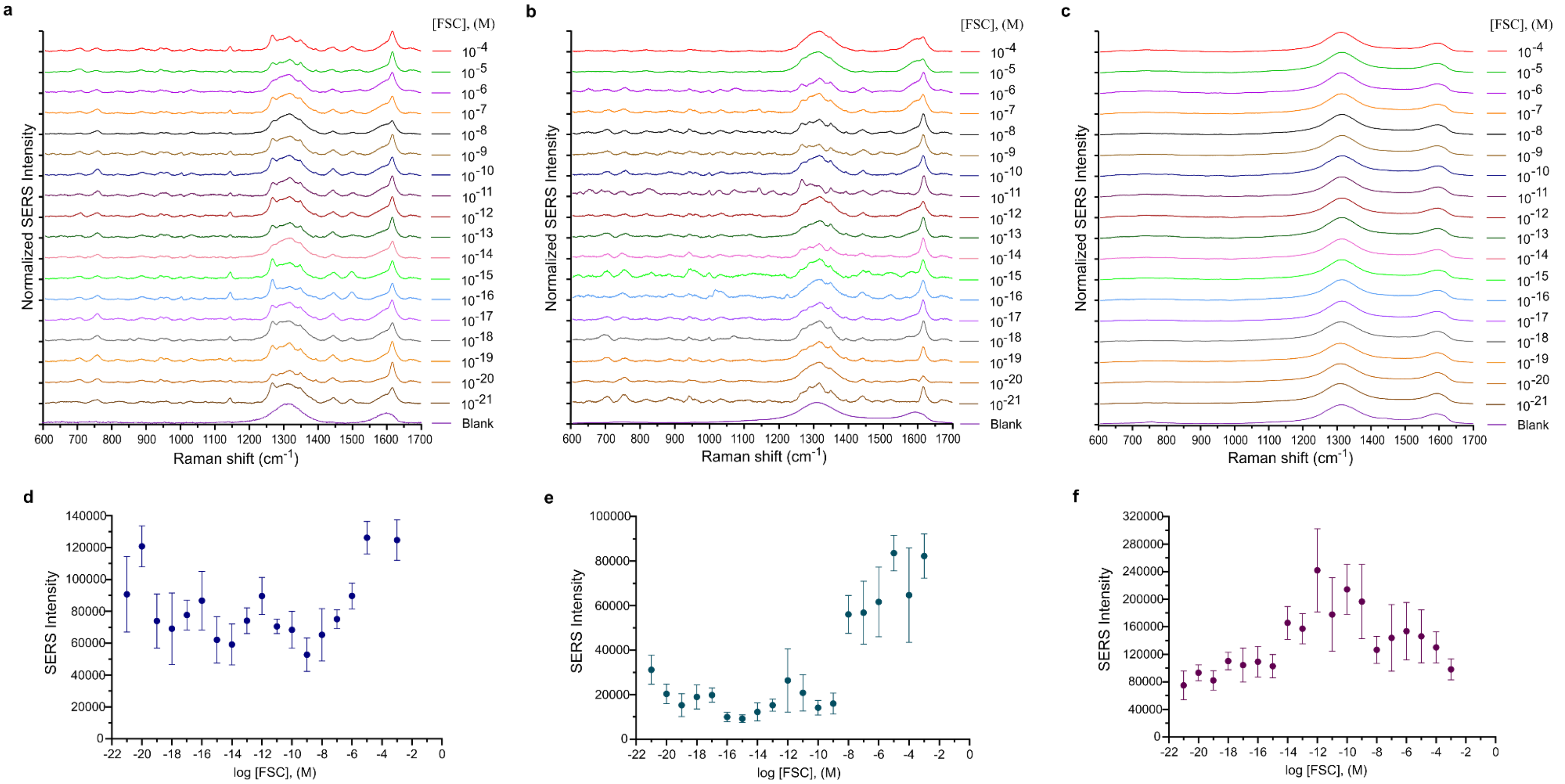

Figure S10. SERS analysis of FSC at several concentrations. Different SERS substrates were employed, (**a**) BC/GO35/AuNRs, (**b**) BC/GO80/AuNRs and (**c**) BC/GO125/AuNRs. Each spectrum represents the mean of ten spectra recorded on random sites of the corresponding SERS substrate. The spectra were obtained with an excitation wavelength of 785 nm; laser power, 0.08 mW; size of the spot, 1.2 µm; exposure time, 2 s; number of acquisitions, 10. The resulting SERS intensity of the peak at the Raman shift of 1616 cm^{-1} in BC/GO35/AuNRs (**d**), BC/GO80/AuNRs (**e**) and BC/GO125/AuNRs (**f**), respectively.

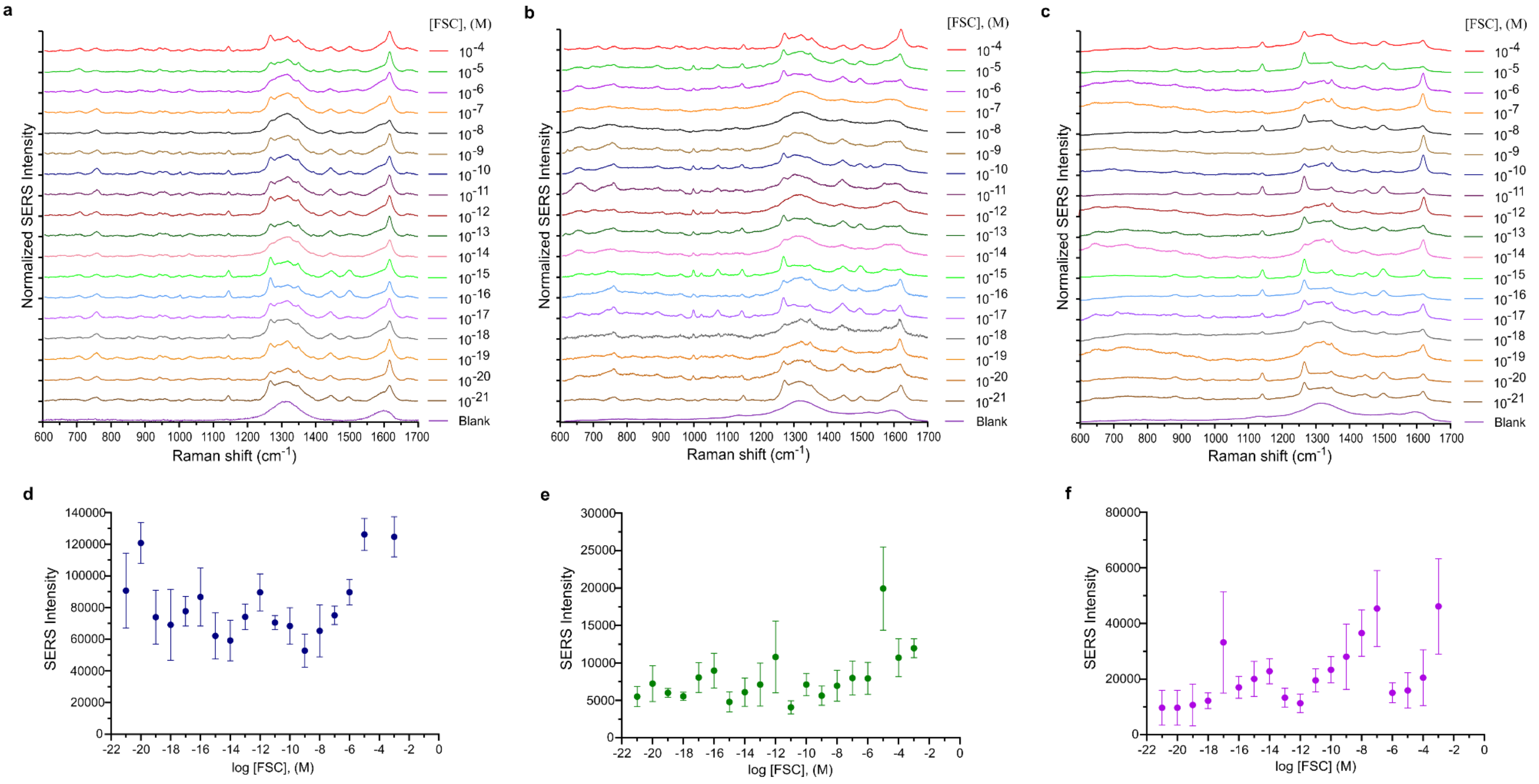

Figure S11. Analysis of repeatability and signal intensity robustness. (**a-c**) SERS spectra of FSC at different concentrations, obtained with 3D-POWER (BC/GO35/AuNRs) fabricated in different batches. **a.** batch 1, **b.** batch 2 and **c.** batch 3. Each spectrum represents the mean of ten spectra recorded on random sites of the corresponding SERS substrate. The spectra were obtained with an excitation wavelength of 785 nm; laser power, 0.08 mW; size of the spot, 1.2 µm; exposure time, 2 s; number of acquisitions, 10. **d-f.** The resulting SERS intensity of the peak at the Raman shift of 1616 cm^{-1} in batch 1 (**d.**), batch 2 (**e.**) and batch 3 (**f.**), respectively.

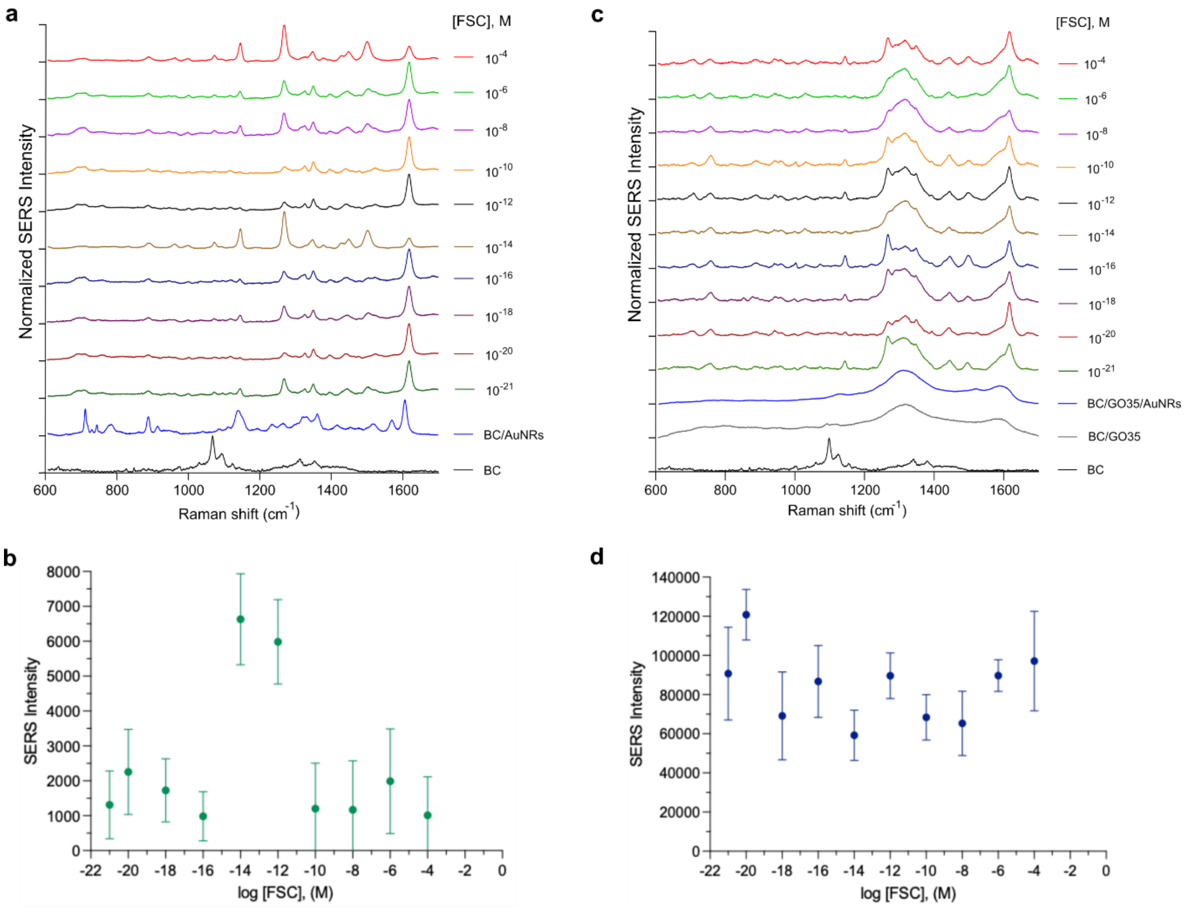

Figure S12. SERS spectra of FSC at several concentrations analyzed with different SERS substrates. **a.** FSC analysis using BC/AuNRs. **c**. FSC analysis using BC/GO35/AuNRs. Each spectrum represents the mean of fifty spectra recorded throughout 2500 μm^2 of the corresponding SERS substrate. The spectra were obtained with an excitation wavelength of 785 nm; laser power, 0.08 mW; size of the spot, 1.2 μm; exposure time, 2 s; number of acquisitions, 10. The resulting SERS intensity of the peak at the Raman shift of 1616 cm^{-1} in BC/AuNRs (**b.**) and BC/GO35/AuNRs (**d.**), respectively.

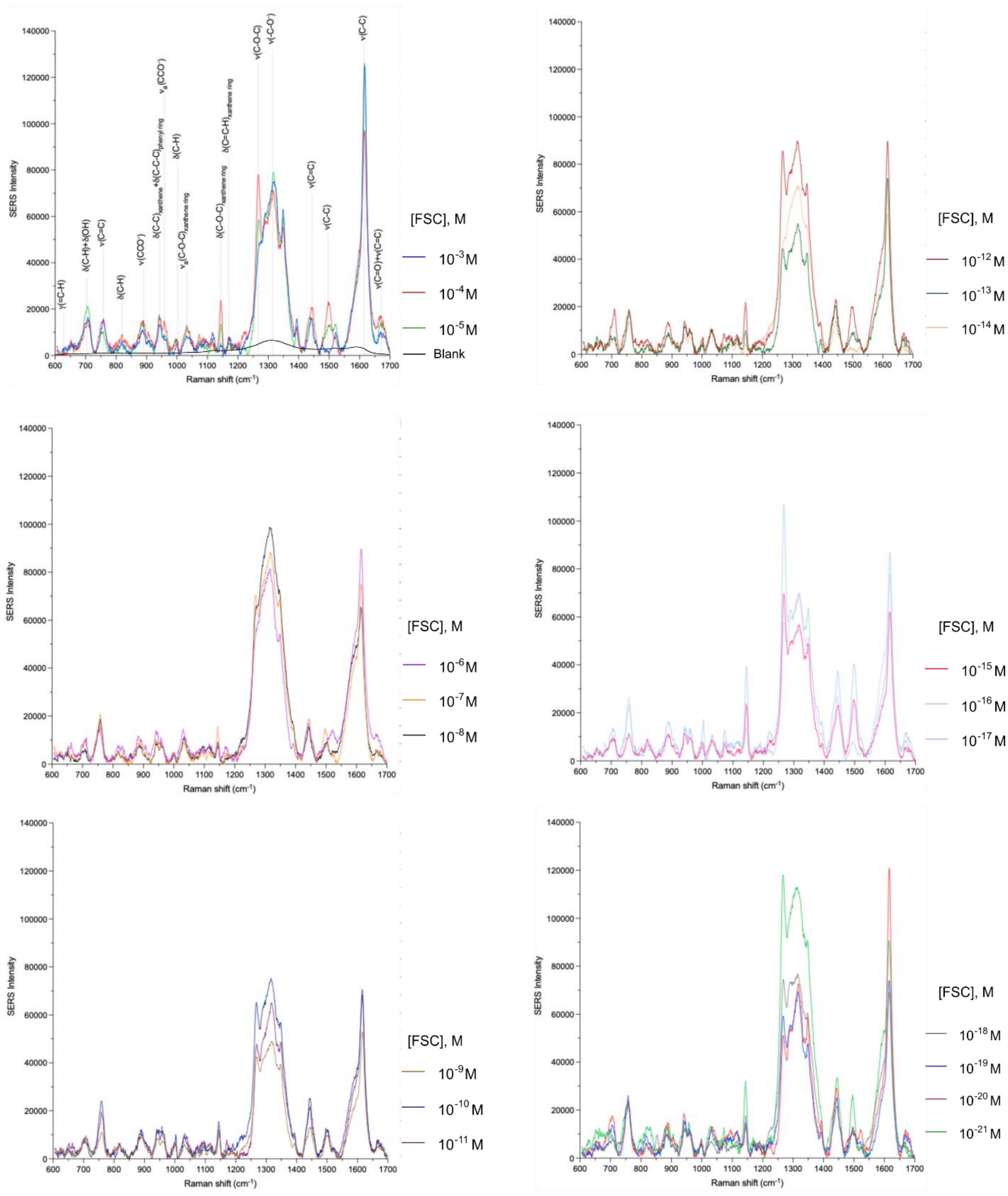

Figure S13. Detailed SERS spectra of FSC analyzed at different concentrations using 3D-POWER (BC/GO35/AuNRs). Each spectrum represents the mean of fifty spectra recorded on 2500 µm^2 of the corresponding SERS substrate. The spectra were obtained through excitation wavelength, 785 nm; laser power 0.08 mW; size of the spot = 1.2 µm; exposure time, 2 s and number of acquisitions, 10.

a

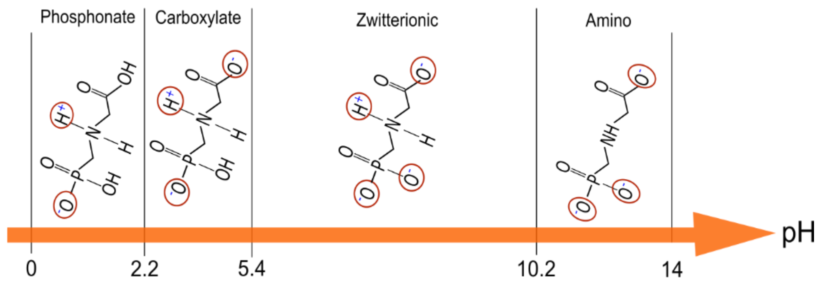

b

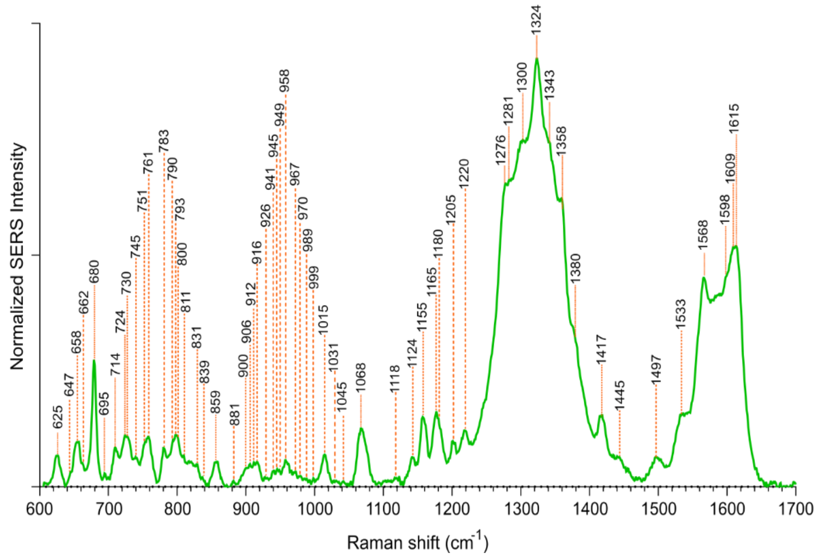

Figure S14. a. Species predominance diagram of GLY, **b.** SERS spectra facilitated by 3D-POWER. The SERS substrate (BC/GO35/AuNRs) was incubated overnight in 1 mL of GLY concentrated at 10^{-9} M. The spectrum represent the mean of fifty spectra recorded on 2500 μm^2 of the corresponding SERS substrate obtained through: excitation wavelength, 785 nm; laser power 0.08 mW; size of the spot = 1.2 μm; exposure time, 2 s. number of acquisitions, 10.

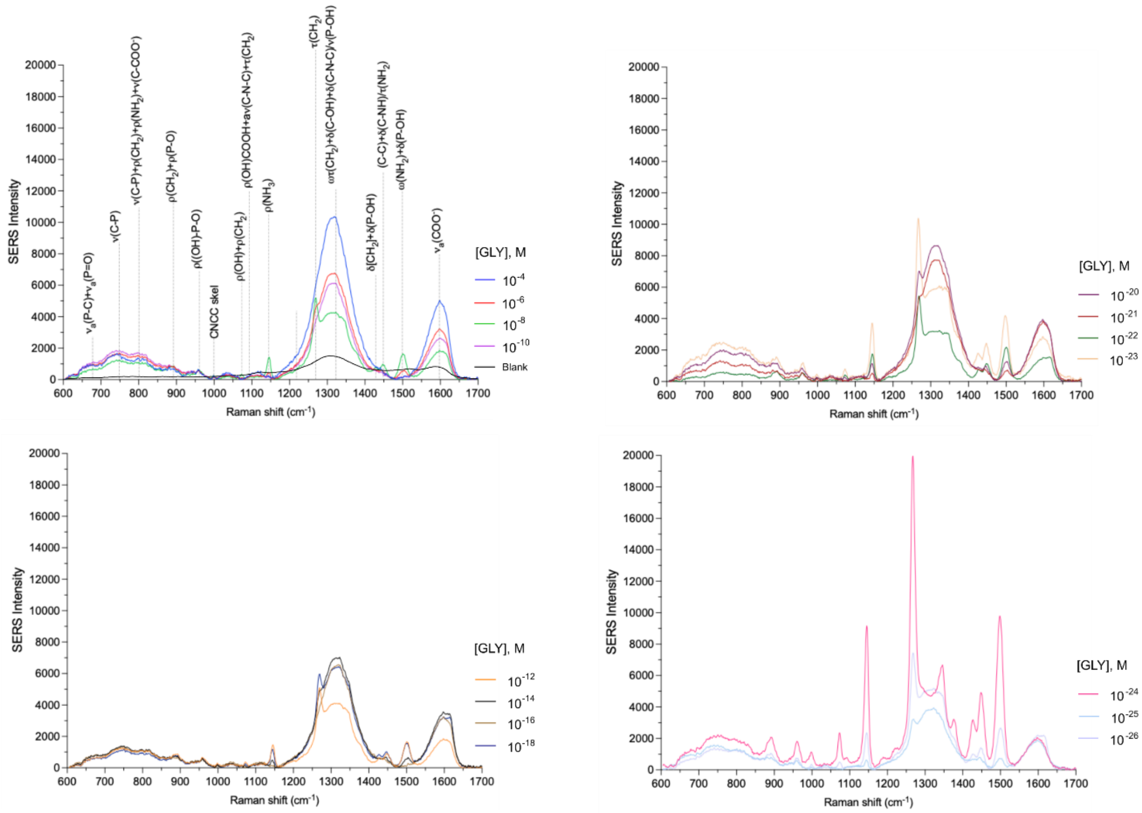

Figure S15. Detailed SERS spectra of GLY analyzed at different concentrations using 3D-POWER (BC/GO35/AuNRs). Each spectrum represents the mean of fifty spectra recorded on 2500 μm^2 of the corresponding SERS substrate. The spectra were obtained through: excitation wavelength, 785 nm; laser power 0.08 mW; size of the spot = 1.2 μm; exposure time, 2 s and number of acquisitions, 10.

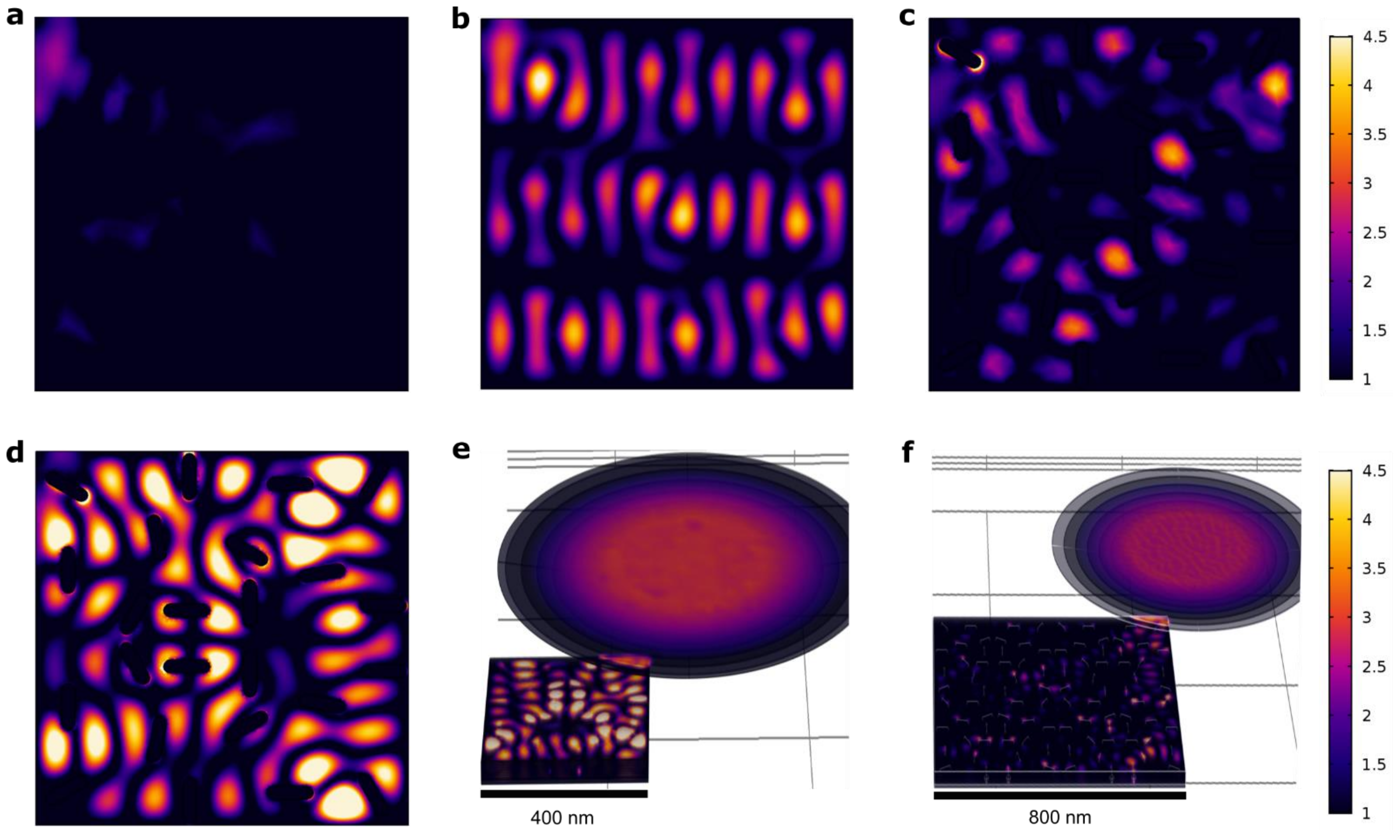

Figure S16. In silico experiments were performed to investigate the light transport capability of the materials composing 3D-POWER. The simulations were performed by establishing the pumped region at the upper right corner of the corresponding material. **a**. Simulation of a plane depicting the light transport capabilities of BC. **b**. Simulation of a plane depicting the light transport capabilities of BC/GO. **c**. Simulation of a plane depicting the plasmonic transmission of BC/AuNR. **d**. Simulation of a plane depicting the plasmonic transmission of 3D-POWER (BC/GO35/AuNR). **e**-**f**. 3D simulations depicting the plasmonic field transport capabilities of 3D-POWER throughout different volumes.

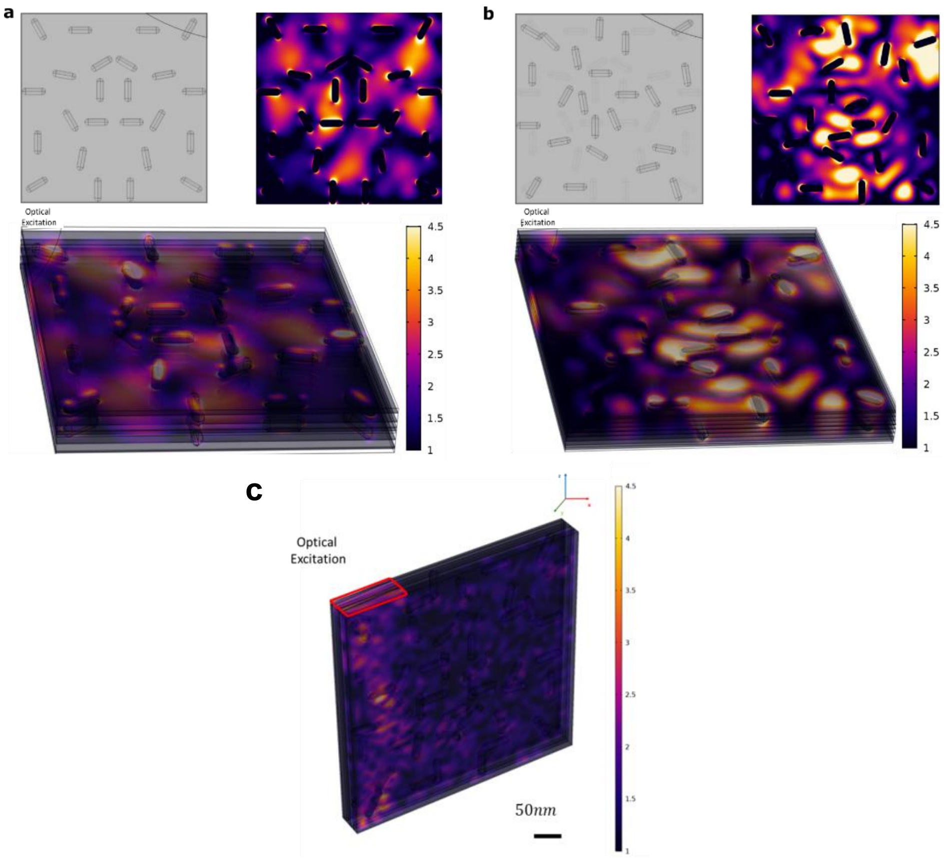

Figure S17. 3D simulations (x = 800 nm, y = 800 nm and z = 50 nm) revealed that 3D-POWER was able to capture light in a small region (5×10^5 nm^3) and perform plasmonic field transmission, thereby generating a plasmonic web throughout the simulated volume. AuNRs were randomly placed. **a.** 3D simulation highlighting plasmonic transmission at the bottom of the simulated volume (excitation perpendicular to the self-assembled layers of 3D-POWER). **b.** 3D simulation highlighting plasmonic transmission at the top of the simulated volume (excitation perpendicular to the self-assembled layers of 3D-POWER). **c**. 3D-*In silico* experiment demonstrating highly efficient transport of surface plasmon polaritons in 3D-POWER under an excitation (top corner-vertical) that is parallel to the self-assembled layers of 3D-POWER.

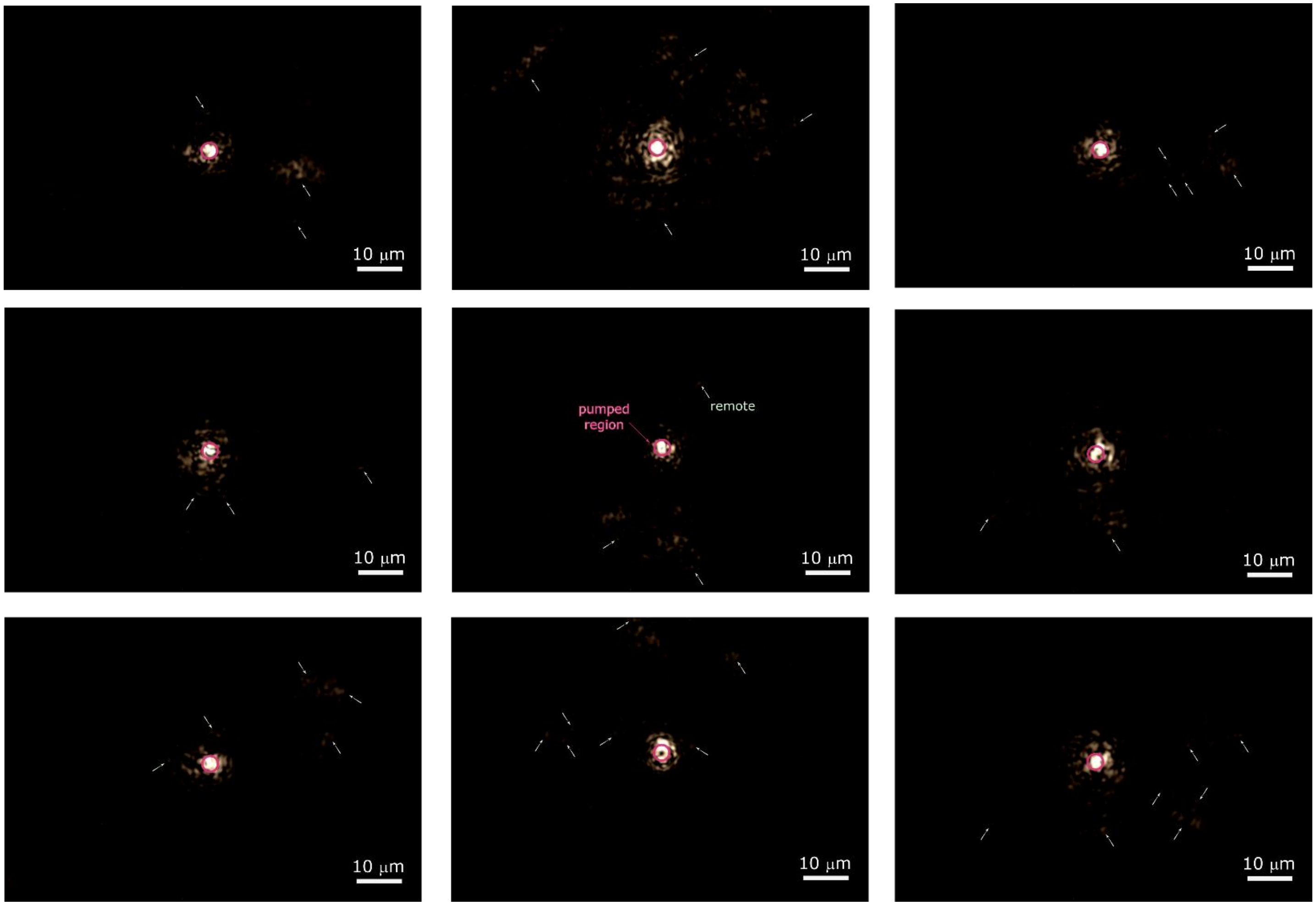

Figure S18. Dark field images of 3D-POWER demonstrating bright emissions from plasmonic nanoparticles beyond the laser spot. The respective pink circle shows the pumping region and the arrows point out the remote emission of light (beyond the laser spot). Laser wavelength, 785 nm; laser power 0.08 mW; size of the spot = 1.2 µm.

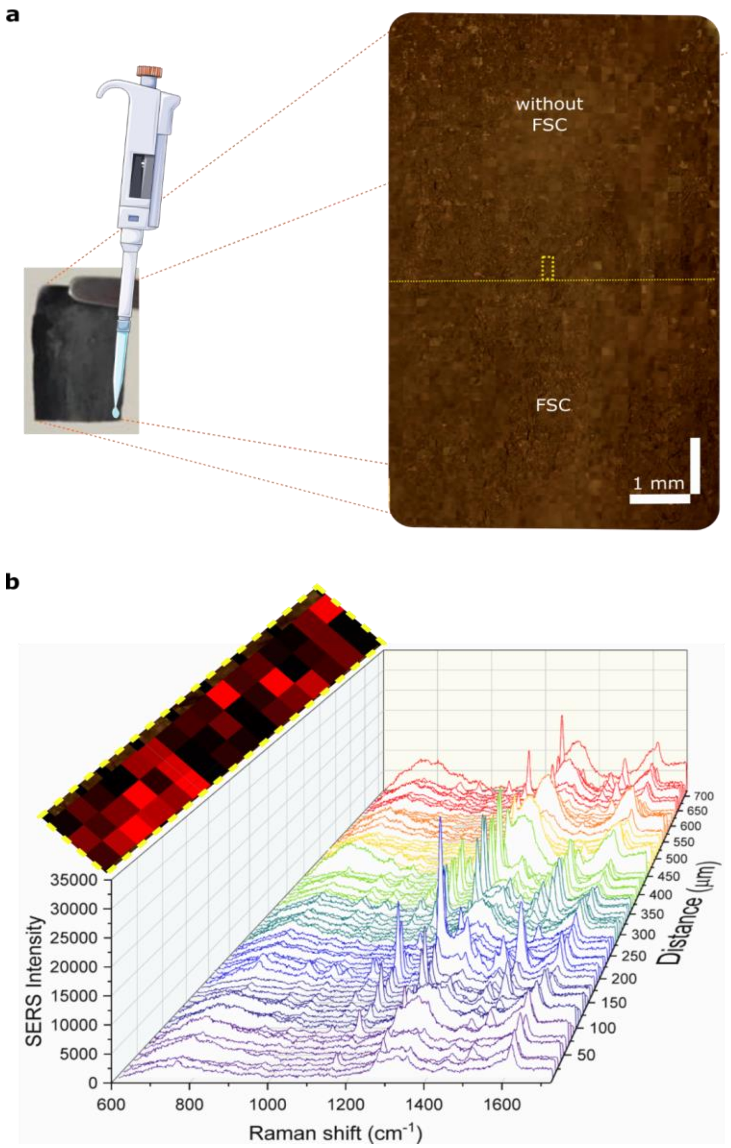

Figure S19. Remote SERS experiment using a rectangular piece of 3D-POWER. **a**. 0.5 µL of FSC concentrated 10^{-9} M (or $9.5X10^{8}$ molecules) were drop casted onto the corner of the substrate. **b**. A Raman mapping in the frontier of the substrate that was not reached by the sample was performed. Signal intensities depicted in the Raman mapping correspond to the intensity at the Raman shift of 1500 cm^{-1}. The spectra were obtained through excitation wavelength, 785 nm; power 0.08 mW; size of the spot = 1.2 µm; exposure time, 2 s; number of acquisitions 10.

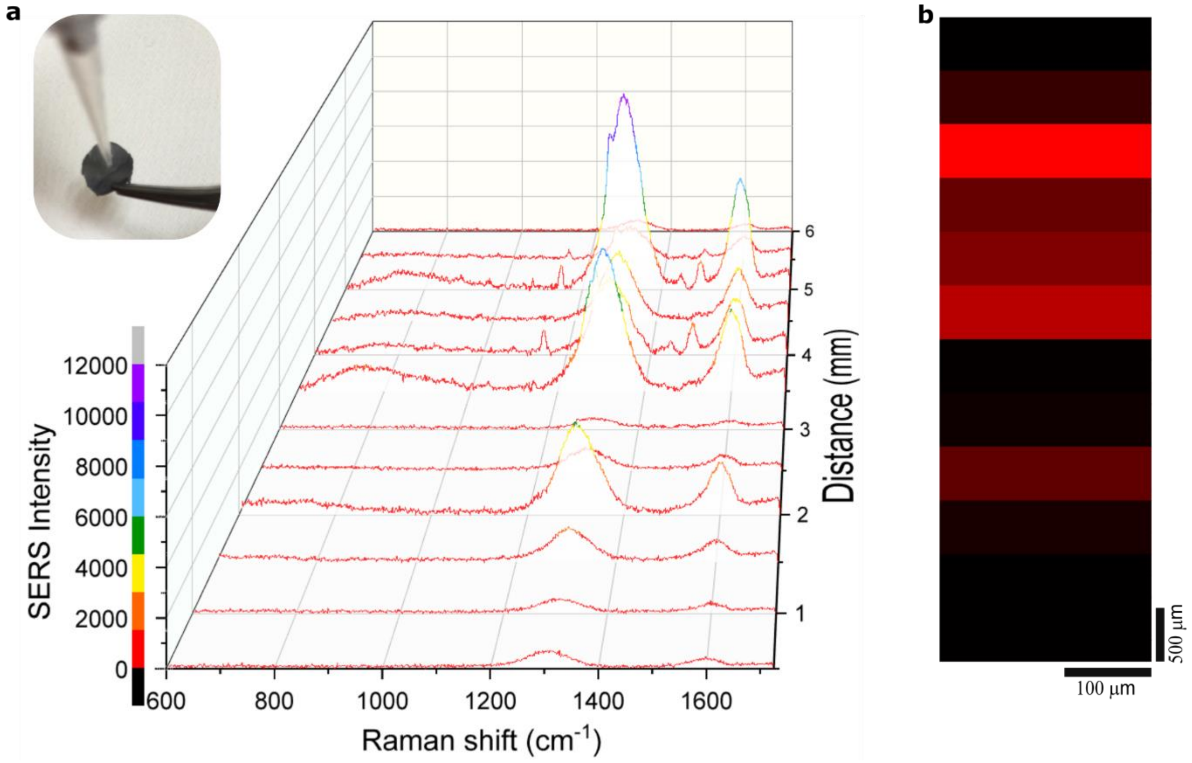

Figure S20. a. 0.1 µL of FSC concentrated at 10^{-16} M (c.a. 10 molecules) were drop casted onto the center of a circular piece of 3D-POWER. Raman mapping with a resolution of 500 µm per step was recorded throughout the diameter (6 mm) of the substrate. **b**. Signal intensities depicted in the Raman mapping correspond to the intensity at the Raman shift of 1500 cm^{-1}. The spectra were obtained through excitation wavelength, 785 nm; power 0.08 mW; size of the spot = 1.2 µm; exposure time, 2 s and number of acquisitions, 10.

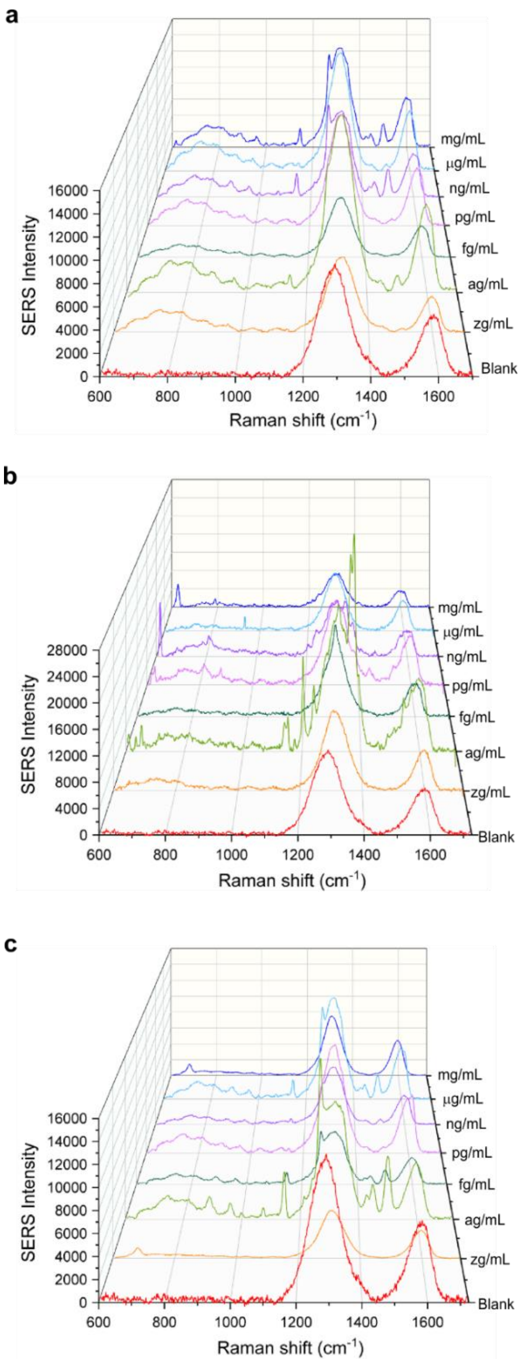

Figure S21. Corn analysis. **a**. SERS spectra of commercially available white cornmeal, incubated at different concentrations. **b.** SERS spectra of commercially available blue cornmeal, incubated at different concentrations. **c.** SERS spectra of non-commercially available cornmeal incubated at different concentrations. Each spectrum represent the means of at least one hundred spectra recorded in an area of 2500 μm^2 of the corresponding SERS substrate. The spectra were obtained through an excitation wavelength of 785 nm; laser power 0.08 mW; size of the spot = 1.2 µm; exposure time, 2 s and number of acquisitions, 10.

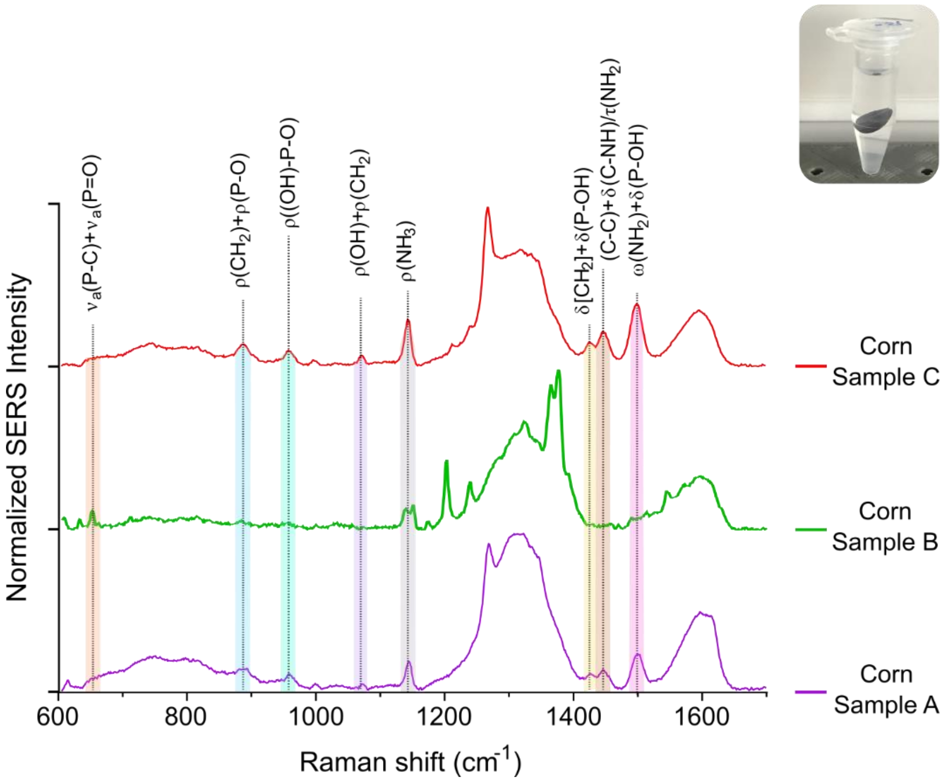

Figure S22. Pesticide detection using 3D-POWER as SERS substrate in commercially available cornmeal (white corn: Sample A; blue corn: Sample B) and non-commercially available white cornmeal (Sample C). The samples were diluted in the order of attograms (10^{-18}) per milliliter. Each spectrum represent the average of at least one hundred spectra recorded in an area of 2500 μm^2 of the corresponding SERS substrate. The spectra were obtained through an excitation wavelength of 785 nm; laser power, 0.08 mW; size of the spot, 1.2 µm; exposure time, 2 s and number of acquisitions, 10.

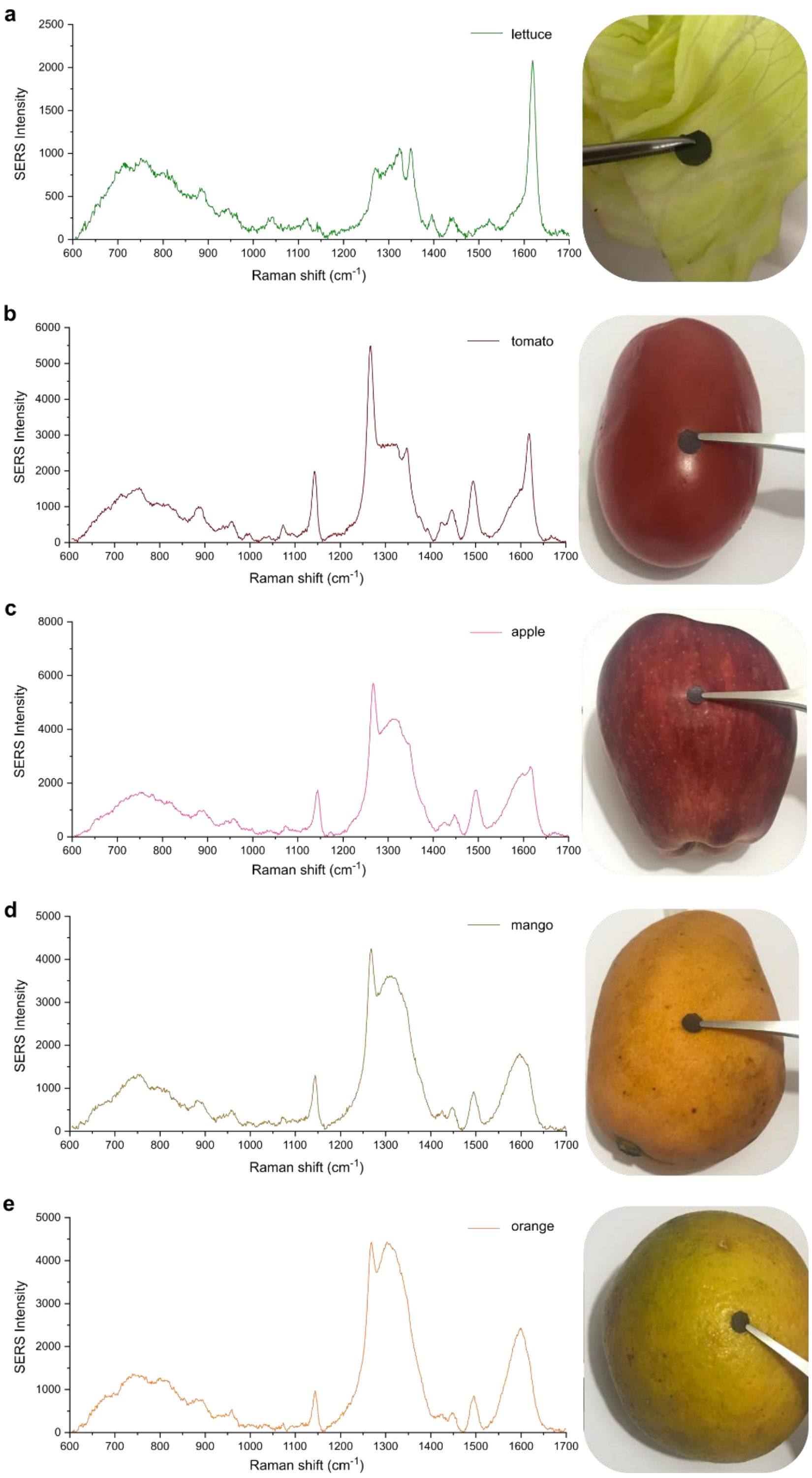

Figure S23. Food analysis. **a.** lettuce, **b.** tomato, **c.** apple, **d.** Mango and **e.** Orange. Each spectrum represent the average of at least 60 spectra recorded in an area of 2500 µm^2 of the corresponding SERS substrate. The spectra were obtained through an excitation wavelength of 785 nm, laser power, 0.8 mW; exposure time, 2 s; size of the spot, 1.2 µm and number of acquisitions, 10.

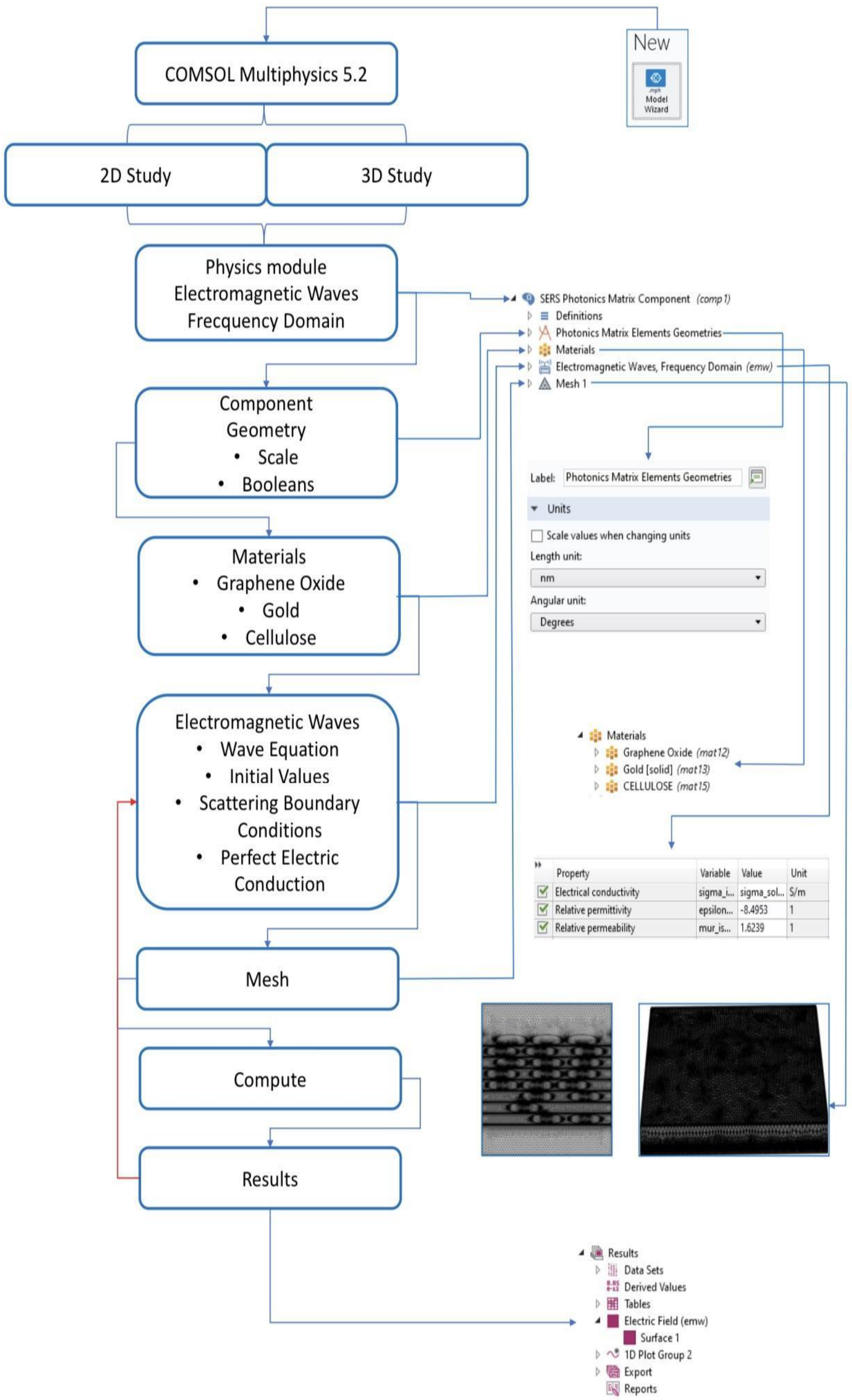

Scheme S3. Flowchart followed in the *in silico* experiments.

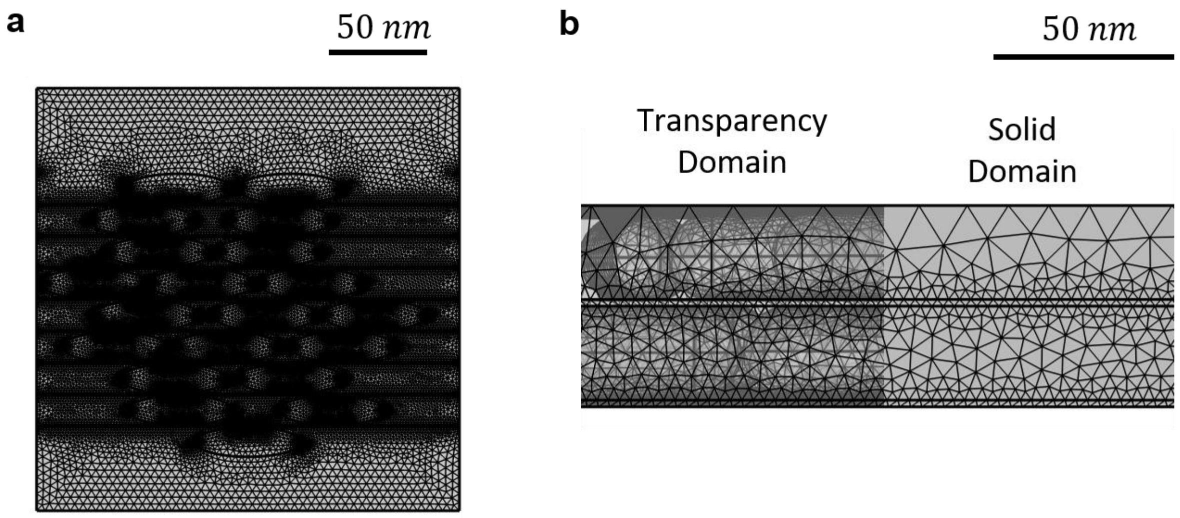

Scheme S4. a. Graphical representation of the meshing employed in simulations representing the electromagnetic enhancement offered by the studied materials. **b.** Graphical representation of the meshing employed in simulations representing the plasmonic transport offered by the studied materials.

Table S1. (Nano)metrology and physicochemical features of each component of 3D-POWER

Component	(Nano)metrology	Surface Chemistry / Physicochemical features	Supplier
Bacterial Nanocellulose	Average size of the fiber is ≈45 ± 10 nm, length of greater than 10 µm	C–OH groups<br>Crystallinity of ≈82%, crystallite size of ≈6.3 nm, tensile strength of ≈345 MPa, Young's modulus of ≈17.3 GPa and strain-at-break of ≈7%.	Nano Novin Polymer Co. (Sari, Iran).
Graphene Oxide (GO)	Aqueous suspension of GO monolayer, average lateral size of ≈500 nm.	C–OH, C–O–C, C=O, and O=C–OH groups<br>Carbon/oxygen ratio around 1.	Angstrom Materials, Inc. (Dayton, OH, USA).
Gold nanorods (AuNRs)	Average length ≈50 ± 10 nm<br>Average diameter ≈14 ± 3 nm	Decorated with cetyltrimethylammonium bromide (CTAB)	Synthesized in the laboratory
3D-POWER (BC/GO35/AuNRs)	Average thickness ≈ 10 µm	C–OH, C–O–C, C=O, and O=C–OH groups	Synthesized in the laboratory

Table S2. UV-Vis features of the explored SERS substrates.

Nanocomposite	Synthesis condition		Experimental results			
	[GO] (µg mL^{-1})	[AuNRs] (mM)	Maximum absorbance	λ (nm)		
BC (blank)	--	--	0.0000	--	--	--
BC/GO20	20	0	0.2933	300	--	--
BC/GO35	35	0	0.5243	300	--	--
BC/GO50	50	0	0.7357	300	--	--
BC/GO65	65	0	0.9403	300	--	--
BC/GO80	80	0	1.2283	300	--	--
BC/GO95	95	0	1.5442	300	--	--
BC/GO105	105	0	1.2690	300	--	--
BC/GO125	125	0	1.6663	300	--	--
BC/GO150	150	0	1.8497	300	--	--
			Maximum absorbance	W_{trans} (nm)	Maximum absorbance	W_{long} (nm)
AuNRs suspension			0.2977	516	0.3907	705
BC/AuNRs	0	0.7	0.2983	518	0.3958	704
BC/GO20/AuNRs	20	0.7	0.2753	518	0.6510	678
BC/GO35/AuNRs	35	0.7	0.4970	518	0.9843	676
BC/GO50/AuNRs	50	0.7	0.3707	520	0.8120	676
BC/GO65/AuNRs	65	0.7	0.3150	520	0.6267	672
BC/GO80/AuNRs	80	0.7	0.4340	524	1.0243	672
BC/GO95/AuNRs	95	0.7	0.4417	520	0.6727	674
BC/GO105/AuNRs	105	0.7	0.5087	520	0.9577	668
BC/GO125/AuNRs	125	0.7	0.7530	518	1.7543	662
BC/GO150/AuNRs	150	0.7	0.4047	520	0.6967	664

λ, wavelength; BC, bacterial nanocellulose; GO, graphene oxide; AuNRs, gold nanorods, W_{trans}, wavelength of the AuNRs transversal plasmon; W_{long}, wavelength of the AuNRs longitudinal plasmon.

Table S3. Particle density in the studied biohybrids.

Nanocomposite	[GO] (μg mL^{-1})	AuNRs average size (nm)	AuNRs number [NRs per $μm^2$] superficial view	AuNRs number [NRs per $μm^2$] transversal view
AuNRs suspension		50±10 X 14± 3	--	--
BC/AuNRs	0	45±10 X 14± 3	105 ± 19	358 ± 29
BC/GO/AuNRs 35	35	49±5 X 15 ± 3	150 ± 14	497 ± 25
BC/GO/AuNRs 80	80	49±5 X 14±5	207 ± 37	402 ± 61
BC/GO/AuNRs 125	125	48±12 X 14± 3	493 ± 72	270 ± 37

BC, bacterial nanocellulose; GO, graphene oxide; AuNRs, gold nanorods. The number of AuNRs per $μm^2$ was determined by analyzing SEM images (superficial and transversal views, respectively).

Table S4 Mean values and ranges of the resulting electromagnetic enhancement factors (γ) in the explored materials.

Nanocomposite	Plane	Intensity		Gamma ($\gamma \approx \frac{|E_{loc}|^4}{|E_0|^4}$)	
		Mean value	Range	Mean value	Range
BC/AuNRs	Horizontal	3.2 ± 1.1	2 to 7	110	11 to 2841
BC/AuNRs	Frontal	4.0 ± 1.5	1 to 8	249	4 to 3598
BC/GO35/AuNRs	Horizontal	3.4 ± 0.7	2 to 6	132	25 to 1376
BC/GO35/AuNRs	Frontal	8.1 ± 2.4	3 to 14	4226	130 to 42522

BC, bacterial nanocellulose; GO, graphene oxide; AuNRs, gold nanorods.

Table S5 Estimation of the number of FSC molecules.

log [FSC]	Experimental concentration, (mol/L)	Moles number, η	Molecules number
-3	1.81E-03	1.81E-05	1.09E+19
-4	1.81E-04	1.81E-07	1.09E+17
-5	1.81E-05	1.81E-08	1.09E+16
-6	1.81E-06	1.81E-09	1.09E+15
-7	1.81E-07	1.81E-10	1.09E+14
-8	1.81E-08	1.81E-11	1.09E+13
-9	1.81E-09	1.81E-12	1.09E+12
-10	1.81E-10	1.81E-13	1.09E+11
-11	1.81E-11	1.81E-14	1.09E+10
-12	1.81E-12	1.81E-15	1.09E+09
-13	1.81E-13	1.81E-16	1.09E+08
-14	1.81E-14	1.81E-17	1.09E+07
-15	1.81E-15	1.81E-18	1.09E+06
-16	1.81E-16	1.81E-19	1.09E+05
-17	1.81E-17	1.81E-20	1.09E+04
-18	1.81E-18	1.81E-21	1087.30
-19	1.81E-19	1.81E-22	108.73
-20	1.81E-20	1.81E-23	10.87
-21	1.81E-21	1.81E-24	1.09

Table S6. Statistical analysis of the explored SERS substrates. FSC was employed as a model analyte.

	Nanocomposite								
	BC/GO35/AuNRs			BC/GO80/AuNRs			BC/GO125/AuNRs		
[FSC], M	Raman Intensity	SD	CV	Raman Intensity	SD	CV	Raman Intensity	SD	CV
1.00E-03	124720	12669	0.10	82234	9921	0.12	98262	15233	0.16
1.00E-04	97134	25409	0.26	64722	21205	0.33	130105	22612	0.17
1.00E-05	126210	10162	0.08	83537	8013	0.10	146131	38391	0.26
1.00E-06	89737	8105	0.09	61673	15644	0.25	153529	41974	0.27
1.00E-07	75094	5884	0.08	56828	14121	0.25	143839	48267	0.34
1.00E-08	65331	16441	0.25	56057	8546	0.15	126496	19759	0.16
1.00E-09	52783	10433	0.20	16017	4691	0.29	196538	53842	0.27
1.00E-10	68349	11585	0.17	14163	3285	0.23	214402	36158	0.17
1.00E-11	70539	4529	0.06	20860	8166	0.39	177911	53590	0.30
1.00E-12	89654	11685	0.13	26422	14208	0.54	242038	60383	0.25
1.00E-13	74091	8096	0.11	15294	2722	0.18	157241	21997	0.14
1.00E-14	59189	12842	0.22	12289	4106	0.33	165556	23924	0.14
1.00E-15	62106	14570	0.23	9243	1743	0.19	102855	16914	0.16
1.00E-16	86713	18356	0.21	9988	2139	0.21	109239	22169	0.20
1.00E-17	77744	9354	0.12	19818	3266	0.16	104433	24776	0.24
1.00E-18	69116	22415	0.32	18980	5418	0.29	110198	12755	0.12
1.00E-19	73926	17025	0.23	15330	1157	0.08	82274	14132	0.17
1.00E-20	120759	12943	0.11	20401	4367	0.21	93243	11559	0.12
1.00E-21	90716	23644	0.26	31168	6527	0.21	75022	20881	0.28
		Mean	0.17 ± 0.08			0.24 ± 0.11			0.21 ± 0.07

BC, bacterial nanocellulose; GO, graphene oxide; AuNRs, gold nanorods, FSC, fluorescein; SD, standard deviation; CV, coefficient of variation. Raman intensities were measured at the Raman shift 1616 cm^{-1}.

Table S7. Assessment of the behavior of BC/GO35/AuNRs in different batches. FSC was employed as a model analyte.

	Batch 1			Batch 2			Batch 3		
[FSC], M	Raman Intensity	SD	CV	Raman Intensity	SD	CV	Raman Intensity	SD	CV
1.00E-03	124720	12669	0.10	11972	1266	0.11	46163	17148	0.37
1.00E-04	97134	25409	0.26	10713	2541	0.24	20517	10029	0.49
1.00E-05	126210	10162	0.08	19943	5562	0.28	15950	6419	0.40
1.00E-06	89737	8105	0.09	7942	2142	0.27	15090	3580	0.24
1.00E-07	75094	5884	0.08	7997	2269	0.28	45365	13687	0.30
1.00E-08	65331	16441	0.25	6957	2068	0.30	36542	8324	0.23
1.00E-09	52783	10433	0.20	5634	1279	0.23	28051	11776	0.42
1.00E-10	68349	11585	0.17	7108	1479	0.21	23364	4720	0.20
1.00E-11	70539	4529	0.06	4088	881	0.22	19565	4150	0.21
1.00E-12	89654	11685	0.13	10795	4791	0.44	11324	3337	0.29
1.00E-13	74091	8096	0.11	7109	2883	0.41	13319	3407	0.26
1.00E-14	59189	12842	0.22	6097	1885	0.31	22839	4532	0.20
1.00E-15	62106	14570	0.23	4787	1342	0.28	20058	6294	0.31
1.00E-16	86713	18356	0.21	8968	2334	0.26	17042	3988	0.23
1.00E-17	77744	9354	0.12	8062	2010	0.25	33194	18281	0.55
1.00E-18	69116	22415	0.32	5538	552	0.10	12205	2823	0.23
1.00E-19	73926	17025	0.23	6010	587	0.10	10694	7545	0.71
1.00E-20	120759	12943	0.11	7236	2393	0.33	9671	6321	0.65
1.00E-21	90716	23644	0.26	5509	1342	0.24	9671	6321	0.65
		Mean	0.17 ± 0.08		Mean	0.26 ± 0.09		Mean	0.37 ± 0.17

BC, bacterial nanocellulose; GO, graphene oxide; AuNRs, gold nanorods, FSC, fluorescein; SD, standard deviation; CV, coefficient of variation. Raman intensities were measured at the Raman shift 1616 cm^{-1}.

Table S8. Vibrational bands observed in the SERS spectra of FSC (diluted in ethanol).

Raman shift in this work (cm^{-1})	Assignation	Reference
629	γ(=C-H)	[1]
654	γ(=C-H)	[1]
698	δ(C-H) + δ(OH)	[1]
713	δ(C-H) + δ(OH)	[1]
758	ν(C=C) for ortho substituted phenyl ring	[1]
801	δ(C-OH) in xanthene ring	[1]
820	δ(C-H)	[1]
830	γ(C-H) for two adjacent hydrogen atoms	[1]
854	γ(C-H)	[1]
870	δ(C-H) equatorial	[1]
885	ν(CCO^-)	[1]
903	γ(C-H)	[1]
942	δ(C-C) of xanthene + δ(C-C-C) of phenyl ring	[1]
957	ν_a(COO^-)	[1]
1000	δ(C-H) in phenyl ring	[2]
1029	ν_a(C-O-C) in xanthene ring	[1]
1074	δ(C-H)	[1]
1094	δ(C-O) in xanthene ring.	[1]
1145	δ(C-O-C) in xanthene ring	[3]
1170	C=C-H bend in xanthene ring	[4]
1191	δ(O=C-OH),	[4]
1224	ν(C-O-C)	[4]
1268	ν_a(C-O-C)	[3]
1314	ν(-C-O^-) conjugated with xanthene ring	[4]
1350	δ(C-H)	[3]
1393	ν(COO^-)	[4]
1445	ν(C=C) in xanthene and phenyl rings	[1]
1500	ν(C-C) in central ring breathing,	[1]
1524	ν_a (COO^-)	[4]
1594	ν(C-C) containing conjugate carbonyl	[4]
1616	ν(C-C) in xanthene skeleton	[5]
1666	ν(C=O) + ν(C=C)	[2]

γ= bending out of plane, δ = bending, ν=stretching, ν_a= asymmetric stretching, vibrational modes were assigned according to existing literature reported by Socrates[1] and Larkin[3].

Table S9. Analytical enhancement factor observed in FSC via 3D-POWER.

[FSC], (M)	Intensity at Raman shift (cm^{-1})				Molecules number (η)	AEF
	758	1145	1445	1616		
10^{-4}	15923	23746	20720	97134	1.09E+17	$2X10^{2}$
10^{-5}	10188	13563	15406	126210	1.09E+16	$2X10^{3}$
10^{-6}	15351	7885	16863	89142	1.09E+15	$2X10^{4}$
10^{-7}	20901	15745	18457	74914	1.09E+14	$2X10^{5}$
10^{-8}	18764	9183	15620	64816	1.09E+13	$2X10^{6}$
10^{-9}	11132	10249	13299	52517	1.09E+12	$2X10^{7}$
10^{-10}	24202	15582	25371	68144	1.09E+11	$3X10^{8}$
10^{-11}	19655	12231	21759	70206	1.09E+10	$2X10^{9}$
10^{-12}	19017	21792	23218	89654	1.09E+09	$3X10^{10}$
10^{-13}	18110	10047	20454	74091	1.09E+08	$2X10^{11}$
10^{-14}	13541	4717	14241	59189	1.09E+07	$2X10^{12}$
10^{-15}	11393	23835	22870	62106	1.09E+06	$3X10^{13}$
10^{-16}	23600	39320	37210	86713	1.09E+05	$4X10^{14}$
10^{-17}	26711	14214	26812	76930	1.09E+04	$3X10^{15}$
10^{-18}	21759	17847	21572	69116	1087.30	$2X10^{16}$
10^{-19}	26064	14650	25102	73857	108.73	$3X10^{17}$
10^{-20}	23886	14017	28728	120759	10.87	$3X10^{18}$
10^{-21}	23871	32087	32970	90120	1.09	$4X10^{19}$

BC, bacterial nanocellulose; GO, graphene oxide; AuNRs, gold nanorods, [FSC], concentration of fluorescein, M, mol/L. The AEF, Analytical Enhanced Factor, was obtained with the intensity in BC/FSC at 10^{-4} M in Raman spectra at 1616 cm^{-1}.

Table S10. Estimation of the number of GLY molecules.

log [GLY]	Experimental concentration, (mol/L)	Moles number, η	Molecules number
-3	5.88E-03	5.88E-01	4E+23
-4	5.88E-04	5.88E-02	4E+22
-5	5.88E-05	5.88E-03	4E+21
-6	5.88E-06	5.88E-04	4E+20
-7	5.88E-07	5.88E-05	4E+19
-8	5.88E-08	5.88E-06	4E+18
-9	5.88E-09	5.88E-07	4E+17
-10	5.88E-10	5.88E-08	4E+16
-11	5.88E-11	5.88E-09	4E+15
-12	5.88E-12	5.88E-10	4E+14
-13	5.88E-13	5.88E-11	4E+13
-14	5.88E-14	5.88E-12	4E+12
-15	5.88E-15	5.88E-13	4E+11
-16	5.88E-16	5.88E-14	4E+10
-17	5.88E-17	5.88E-15	4E+09
-18	5.88E-18	5.88E-16	4E+08
-19	5.88E-19	5.88E-17	4E+07
-20	5.88E-20	5.88E-18	4E+06
-21	5.88E-21	5.88E-19	4E+05
-22	5.88E-22	5.88E-20	4E+04
-23	5.88E-23	5.88E-21	3540.89
-24	5.88E-24	5.88E-22	354.09
-25	5.88E-25	5.88E-23	35.41
-26	5.88E-26	5.88E-24	3.54
-27	5.88E-27	5.88E-25	0.35

SERS substrate: BC/AuNRs or BC/GO35/AuNRs, BC, bacterial nanocellulose; GO, graphene oxide; AuNRs, gold nanorods, [GLY], concentration of glyphosate, M, mol/L.

Table S11. Vibrational bands observed in in the analysis of GLY concentrated at 10^{-9} M (diluted in HPLC grade water).

Theoretical Raman shift (cm^{-1})	Raman shift (cm^{-1}) In this work	Assignation	Reference
629	625	ν(P-C) + δ(N-C-C) + δ(C-O-O)	[6]
639	647	ν(P-C) + δ(N-C-C) + δ(C-O-O)	[7]
659	658	o.p. δ(C=O)	[8]
662	662	o.p. δ(C=O)	[8]
680	680	ν_a(P-C) + ν_a(P=O)	[9]
700	695	ν(P-C)	[10]
714	714	ν_a(P-CH_2)	[9]
720	724	δ(N-H) + ν(C-P)	[7]
731	730	δ(N-H) + ν(C-P)	[11]
747	745	ν_a(O-P-O)	[9]
750	751	ν(P-C)	[10]
761	761	ν(P-C), δ(N-H) + ρ(CH_2) + ν(P-OH)	[6,11]
783	783	ν(P-C + ρ(CH_2) + ρ(NH_2) + ν(C-COO)	[6]
790	790	o.p. ν(C=O)	[8]
794	793	ρ(N-H),	[9]
799	800	ν(P-C) + ρ(CH_2) + ρ(NH_2) + ν(C-COO)	[7]
819*	811	ρ(CH_2) + ν(P-OH)	[6]
830	831	ν(P-OH)	[7]
839	839	ρ(CH_2) + δ(N-H) + ν(C-C)	[11]
857	859	ρ(P-O) + ρ(OH)HO-P=O	[9]
880	881	ρ(CH_2) + ρ(P-O)	[9,11]
885	900	ρ(CH_2)	[7]
906	906	C-N-C-C skel	[6]
911	912	ν(P-O)	[9]
917	916	C-N-C-C skel	[7]
927	926	ν(P-O) + ρ(OH), ρ(CH_2)	[7,9]
942	941	ρ(OH-P)	[6]
944	945	ρ(CH_2)	[9]
948	949	ν_s(PO_3) + ν(P-C)	[6]
961	958	ρ(OH)-P-O	[9]
968	967	ρ(CH_2) + δ(OH)	[11]
978	970	ν_s(PO_3) + τ(CH_2) + ρ(NH_2) + CNCC skel	[6]
988	989	ρ(OH)HOP=O	[9]
999	999	CNCC skel	[6]
1017	1015	sν(C-H) + ρ(OH)	[9]
1031	1031	ρ(OH)HOP=O	[9]
1047	1045	ν(C-N) + ν(C-OH)	[11]
1050	1068	ρ(OH) + ρ(CH_2)	[9]
1074	1072	ρ(OH)COOH + ν_a(C-N-C) + τ(CH_2)	[9]
1123	1118	ν_a(P-OH)	[6]
1128	1124	ν_a(C-N-C)	[9]
1151	1155	ρ (NH_3)	[6]
1167	1165	δ(CH_2 + NH_2+CH_2) + ν(COH)	[7]
1174	1180	ρ(OH)COOH + τ(CH_2)	[9]
1196	1205	δ(CH_2 + NH_2+CH_2) + ν(COH) + ν(CN)	[7]
1226	1220	ρ(NH) + τ(CH_2), δ(CH_2 + NH_2+CH_2) + ν(CN) + (COH)	[6,9]
1242	1246	ν(P-OH) + τ(CH_2) + ν(C-OH) +δ(C-N-C), ω(CH_2)	[6,11]
1271	1276	τ(CH2)	[9]
1280	1281	ν(P-C) + ν(P-OH) + ω(CH_2)	[6]
1303	1300	ω(CH_2) + ωτ(CH_2) + δ(C-OH) + ν(P-C)	[6]
1327	1325	ωτ(CH_2) + δ(C-OH) + δ(C-N-C)/ ν(P-OH)	[6]
1340	1343	ω(CH2)	[11]
1364	1358	γ(N-CH_2) + δ(NH)	[12]
1380	1380	δ(CH_2) + ρ(C-C-OH)	[6]
1431	1417	δ[CH_2] + δ(P-OH)	[6]
1445	1445	(C-C) + δ(C-NH)/ τ(NH_2)	[6]
1485	1497	ω(NH_2) + δ(P-OH)	[6]
1534*	1533	δ(NH_3)	[6]
1567	1568	δ(NH_2)	[6]
1589	1598	ν_a(COO^-)	[12]
1611	1609	ν(C=O)	[9]
1619	1615	δ(NH_2^+)[a]	[11]

ν= stretching, ν_a = asymmetric stretching, δ= bending, τ = twisting, ω = wagging, ρ = rocking, [a] the band at 1615 cm^{-1} is assigned to δ(NH_2^+) of the zwitterionic species.

Table S12. Analytical enhancement factor for GLY via 3D-POWER.

[GLY], (M)	Intensity at Raman shift				Molecules number (N)	AEF
	880 cm^{-1}	970 cm^{-1}	1417 cm^{-1}	1500 cm^{-1}		
10^{-4}	811	315	1114	150	4E+22	$1X10^{1}$
10^{-6}	860	316	697	527	4E+20	$4X10^{3}$
10^{-8}	796	347	571	1641	4E+18	$1X10^{6}$
10^{-10}	937	359	671	142	4E+16	$1X10^{7}$
10^{-12}	820	379	418	1669	4E+14	$1X10^{10}$
10^{-14}	754	266	633	579	4E+12	$4X10^{11}$
10^{-16}	662	277	734	130	4E+10	$9X10^{12}$
10^{-18}	733	236	676	1573	4E+08	$1X10^{16}$
10^{-20}	1159	440	908	1272	4E+06	$9X10^{17}$
10^{-21}	744	258	856	683	4E+05	$5X10^{18}$
10^{-22}	528	232	429	2191	4E+04	$2X10^{20}$
10^{-23}	1438	495	1356	4068	4E+03	$3X10^{21}$
10^{-24}	1676	851	1874	9557	4E+02	$7X10^{22}$
10^{-25}	813	272	651	714	4E+01	$5X10^{22}$
10^{-26}	955	361	684	2668	4E+00	$2X10^{24}$
10^{-27}	1311	540	510	260	0.4	$2X10^{24}$

BC, bacterial nanocellulose; GO35, graphene oxide at 35 µg·mL^{-1}; AuNRs, gold nanorods, [GLY], concentration of glyphosate, M, mol/L. The AEF, Analytical Enhanced Factor, was obtained with the intensity in BC/GLY at 10^{-4} M Raman spectra at 1500 cm^{-1}.

Table S13. Material properties considered in the *in silico* experiments.

Material properties	Cellulose		Gold		Graphene oxide	
	Value	Ref.	Value	Ref.	Value	Ref.
μ_r	1.00	[13]*	1.62x10^{-6}	**[14]	1.40	[13,15,16]
ϵ_r	2.34	[17]	-22.00	[14,18–20]	3.00	[21,22]
σ_s [S/cm]	0.05	[23,24]	4X10^{6}	**	2000	[25,26]

ϵ_r, permittivity; μ_r; permeability; σ_s; conductivity.
* This value was calculated by following Equation (2) reported by Dumik and co-workers[13]
** Value was obtained in a database provided by the MIT. (https://www.mit.edu/~6.777/matprops/gold.htm)

Table S14. Vibrational bands observed using 3D-POWER, which were rubbed into the surface of vegetables and fruits.

Sample	Theoretical Raman shift (cm^{-1})	Raman shift (cm^{-1}) In this work	Assignation	Reference
Lettuce	890	890	δ(C-O-O)	[1]
	962	965	γ - nylon 6	[27]
	1130	1148	α and γ - nylon 6	[27]
	1276	1270	γ - nylon 6	[27]
	1327	1330	ωτ(CH_2) + δ(C-OH) + δ(C-N-C)/ν(P-OH)	[6]
	1350	1350	δ(C-H)	[3]
	1379	1396	δ(C-N)	[28]
	1444	1450	ν(CH_2)	[27]
	1508	1500	ρ(CH3), ν(C-N) in thiram	[28]
	1594	1593	ν(C-C), in carbonyl	[1]
	1622	1616	ν(C=N) in TBZ	[29]
Tomato	744	746	δ(N-C-C)	[30]
	889	888	Caroteno	[30]
	964	965	$ρ_{ip}$ (CH_2)	[30]
	1007	1002	ν(C-CH_3)	[30]
	1079	1074	Diphenyl ether, Cuticular wax	[30,31]
	1143	1143	Keto-enol	[31]
	1269	1268	$δ_{ip}$(=C-H) in β-carotene	[30]
	1351	1350	Carotene	[30]
	1441	1432	δ(CH_2) in cuticular wax	[30]
	1455	1455	Xylene in organophosphorus	[31]
	1498	1495	Acetamiprid	[31]
	1596	1595	triazine	[31]
	1622	1618	ν(C=C) in phenolic compounds	[30]
Apple	653	656	$δ_{ip}$(P=S)	[32]
	778	777	$δ_{oop}$(C-H)	[33]
	890	890	δ(C-O-C)	[1]
	900	903	Strobilurin and triazole	[31]
	949	951	Strobilurin and triazole	[31]
	980	985	Strobilurin and triazole	[31]
	1073	1075	Oxadiazine	[31]
	1148	1143	δ(C-N) + ρ(CH_3) in thiram	[34]
	1168	1168	Organophosphorus	[31]
	1266	1266	Oxadiazine	[31]
	1325	1325	Glycine in glyphosate	[31]
	1416	1417	Aryloxyalkanoic	[31]
	1498	1495	Acetamiprid	[31]
	1534	1535	Bipyridylium in diquat	[31]
	1622	1618	ν(C=N) in TBZ	[29]
Mango	622	625	Triazole	[31]
	728	753	δ(N-C-O-C)	[35]
	883	880	ν(P-O-C) in acephate	[35]
	1140	1140	ν(C-N) in fipronil	[31]
	1277	1277	Malathion + xylene in organophoso	[31]
	1321	1321	δ(C-N) in atrazine	[31]
	1450	1450	δ(C-NH) in organophosphorus	[31]
	1498	1498	ω(NH_2) + δ(P-OH) in glyphosate	[6]
	1584	1596	ketone	[35]
Orange	750	750	ν(P=S) in phoxim	[36]
	925	958	ν(CH_3N); ν(C=S) in thiram	[36]
	1092	1071	γ(C-H) in phoxim; ν(C-C) in melathion	[36]
	1144	1142	ν(P-S) in melathion	[36]
	1264	1267	δ(CH_3) in phoxim	[36]
	1303	1302	ω(CH_2) + ωτ(CH_2) + δ(C-OH) + ν(P-C) in glyphosate	[6]
	1437	1425	$ν_a$(CH_3) in phoxim	[36]
	1444	1445	ν(C-O) in malathion	[36]
	1502	1496	ν(C_6H_5) in phoxim	[36]
	1591	1600	ν(C_6H_5) in phoxim	[36]

ν,stretching; $ν_a$, asymmetric stretching; δ, bending; τ, twisting; ω, wagging; ρ, rocking; $_{ip}$, in plane; oop, out of plane.

Table S15. Vibrational bands observed using 3D-POWER substrates, which were impregnated with sweat for ten seconds in the temple of 4 volunteers.

Sweat component	Range or Raman signal (cm^{-1})	Reference
Uric Acid	496	[37]
Lactic acid or Lactate	747, 828, 870, 1045, 1372, 1455,	[38]
Glucose	400, 1058	[39]
Tyrosine	1139, 1388, 1436	[40]
Urea	1003	[40]
Arginine	424	[41]
Histamine	717	[42]
Amino acids	1000-1600	[43]
Eccrine sweat	580-800, 1230-1600	[44]

Table S16. Vibrational bands observed using 3D-POWER substrates, which were rubbed into the surface of the T zone (face) of two volunteers.

Sebum component	Range Raman signal (cm^{-1})	Reference
Aliphatic chains,	250 - 400	[45]
Cholesterol, squalene	400 - 800	[45], [46]
Cholesterol ester	1380 - 1450	[46]
Fatty acid	800 – 1000	[47]
Membrane lipids	1000 – 1200	[45]
Triacylglycerols	1200 - 1380	[47]
*vitamin E	400 - 600	[46]

Table S17. Cost estimation to 3D-POWER fabrication

Step	Reactant		Cost (USD)
BC	BC	Nano Novin Polymer Co	3.28125
BC/GO	GO	Angstrom Materials; Inc	0.00003
BC/GO35/AuNRs	$AgNO_3$	Sigma-Aldrich	0.01551
	CTAB	Sigma-Aldrich	3.19914
	$HAuCl_4$	Sigma-Aldrich	6.66065
	$NaBH_4$	Sigma-Aldrich	0.00091
	AA	Sigma-Aldrich	0.01282
Total			13.17030*
SERS substrate			0.098

BC, Bacterial Nanocelullose; GO, Graphene Oxide; $AgNO_3$, Silver Nitrate; $NaBH_4$, Sodium borohydride; $HAuCl_4$, Hydrogen tetrachloroaurate; CTAB, Hexadecyl trimetylammonium bromide; AA, Ascorbic Acid. *The cost was estimated by considering the weight of each reagent employed in the fabrication of one batch with 15 pieces of cellulose, each piece has nine circular SERS substrates.

Selection of the optimal SERS substrate.

We evaluated the SERS performance of the fabricated substrates by incubating millimetric circles (diameter, 6 mm) of the substrates (BC/AuNRs, BC/GO35/AuNRs, BC/GO80/AuNRs BC/GO125/AuNRs) inside a microtube containing 1 mL of fluorescein (free acid), (FSC, CAS 2321-07-5), as a model analyte, at different concentrations, blanks samples were also incubated in this series of experiments. According with Wang[4] and Batistela[48], FSC has three typical pKa values. Each pH range displays one or more predominant species (see **Figure S8**a). Under these conditions, using ethanol as a solvent, the fingerprint of two main species, phenolate and carboxylate of FSC was observed in the SERS spectra, see Figure S8b.

We firstly investigated the performance of BC/GO125/AuNRs as a SERS substrate. In these series of measurements, the intensity of the D and G Raman bands of GO masked the fingerprint of FSC even at different FSC concentrations, **Figure S9**c. Lower exposure times and number of acquisitions were also explored with similar results. BC/GO35/AuNRs and BC/GO80/AuNRs were also evaluated using different concentrations of FSC, from 10^{-4} M to 10^{-21} M respectively, see Figure S9a-b. In both cases, we realized that we were able to detect extremely low concentrations of FSC, even at the zeptomolar (zM) range (see **Table S6**). However, in general, BC/GO35/AuNRs had the highest Raman intensity, the lowest coefficient of variation (CV, which was about 0.17 ± 0.08), and minimal Raman interference such as fluorescence and shot noise, see Figure S9a-b.

We also compared the SERS performance of BC/GO35/AuNRs in three different batches. In all cases the FSC fingerprint was observable in a concentration range from 10^{-3} M to 10^{-21} M. The batch one showed the lower variability in terms of coefficient of variation (CV is 0.17 ± 0.08), see Figure S10 and Table S7. Hence, batch one was generally employed in this research. We also compared the analytical behavior of BC/GO35/AuNRs with that of BC/AuNRs. Using BC/AuNRs for FSC analysis, the FSC vibrational bands are not fully observed, whereas the blank sample showed vibrational bands associated to CTAB and BC.[1],[49] Hence, the FSC fingerprint was difficult to distinguish at those concentrations below 10^{-16} M. In contrast, BC/GO35/AuNRs offered the capability to analyze the Raman fingerprints of FSC at the zM range, see Table S8 and **Figure S11**.

Motivated by the ultrasensitive character of 3D-POWER, we also analyzed glyphosate (GLY, CAS: 287399-31-9, analytical standard). GLY is one of the most widely used pesticides in in the world, particularly in corn, soybean and sorghum. According with Valle and co-workers[50], "the challenge to detect GLY residue using a simple analytical method is due to its ionic character, high polarity, and solubility in water", and other physicochemical properties. Glyphosate in aqueous solution has two deprotonating mechanisms,[51] see Figure S13. Using 3D-POWER, we analyzed the Raman fingerprint of GLY with high detail and ultrasensitive character, see Figure S13b and S14.

Supporting References

[1] G. Socrates, *Infrared and Raman characteristic group frequencies: tables and charts*, 3. ed., Wiley, Chichester, **2001**.

[2] P. Hildebrandt, M. Stockburger, *J. Raman Spectrosc.* **1986**, *17*, 55.

[3] P. J. Larkin, *Infrared and raman spectroscopy: principles and spectral interpretation*, 2e ed., Elsevier, Amsterdam, **2011**.

[4] L. Wang, A. Roitberg, C. Meuse, A. K. Gaigalas, *Spectrochimica Acta Part A: Molecular and Biomolecular Spectroscopy* **2001**, *57*, 1781.

[5] T. Maeda, T. Nagahara, M. Aida, T. Ishibashi, *J. Raman Spectrosc.* **2008**, *39*, 1694.

[6] J. Ascolani Yael, J. D. Fuhr, G. A. Bocan, A. Daza Millone, N. Tognalli, M. dos Santos Afonso, M. L. Martiarena, *J. Agric. Food Chem.* **2014**, *62*, 9651.

[7] A. Feis, C. Gellini, M. Ricci, L. Tognaccini, M. Becucci, G. Smulevich, *Vibrational Spectroscopy* **2020**, *108*, 103061.

[8] M.-L. Xu, Y. Gao, Y. Li, X. Li, H. Zhang, X. X. Han, B. Zhao, L. Su, *Spectrochimica Acta Part A: Molecular and Biomolecular Spectroscopy* **2018**, *197*, 78.

[9] J. Castillo, C. Rozo, K. Wu, T. Rindzevicius, A. Boisen, *Univ. Sci.* **2021**, *26*, 51.

[10] G. Emonds-Alt, B. Mignolet, C. Malherbe, J.-C. M. Monbaliu, F. Remacle, G. Eppe, *Phys. Chem. Chem. Phys.* **2019**, *21*, 22180.

[11] J. C. S. Costa, R. A. Ando, A. C. Sant'Ana, P. Corio, *Phys. Chem. Chem. Phys.* **2012**, *14*, 15645.
[12] B. Sjöberg, S. Foley, B. Cardey, M. Enescu, *Spectrochimica Acta Part A: Molecular and Biomolecular Spectroscopy* **2014**, *128*, 300.
[13] A. O. Dumik, A. A. Kalenyuk, V. O. Moskaliuk, A. P. Shapovalov, S. I. Futimsky, O. G. Turutanov, V. Yu. Lyakhno, *J. Nano- Electron. Phys.* **2022**, *14*, 02006.
[14] P. B. Johnson, R. W. Christy, *Phys. Rev. B* **1972**, *6*, 4370.
[15] V. Schmiedova, J. Pospisil, A. Kovalenko, P. Ashcheulov, L. Fekete, T. Cubon, P. Kotrusz, O. Zmeskal, M. Weiter, *Journal of Nanomaterials* **2017**, *2017*, 1.
[16] D. A. Komisar, G. M. Krivova, Y. V. Stebunov, D. I. Yakubovsky, G. A. Ermolaev, A. V. Arsenin, V. S. Volkov, *J. Phys.: Conf. Ser.* **2020**, *1461*, 012068.
[17] H. Cheng, Y. Lu, D. Zhu, L. Rosa, F. Han, M. Ma, W. Su, P. S. Francis, Y. Zheng, *Nanoscale* **2020**, *12*, 9471.
[18] P. Stoller, V. Jacobsen, V. Sandoghdar, *Opt. Lett.* **2006**, *31*, 2474.
[19] A. Kheirandish, N. Sepehri Javan, H. Mohammadzadeh, *Sci Rep* **2020**, *10*, 6517.
[20] L. J. Mendoza Herrera, D. M. Arboleda, D. C. Schinca, L. B. Scaffardi, *Journal of Applied Physics* **2014**, *116*, 233105.
[21] F. C. Salomão, E. M. Lanzoni, C. A. Costa, C. Deneke, E. B. Barros, *Langmuir* **2015**, *31*, 11339.
[22] T. Kavinkumar, D. Sastikumar, S. Manivannan, *RSC Adv.* **2015**, *5*, 10816.
[23] P. Khamwongsa, P. Wongjom, H. Cheng, C. C. Lin, S. Ummartyotin, *Composites Part C: Open Access* **2022**, *9*, 100314.
[24] N. Nirmal, M. N. Pillay, M. Mariola, F. Petruccione, W. E. van Zyl, *RSC Adv.* **2020**, *10*, 27585.
[25] J. T. Hong, K. M. Lee, B. H. Son, S. J. Park, D. J. Park, J.-Y. Park, S. Lee, Y. H. Ahn, *Opt. Express* **2013**, *21*, 7633.
[26] Y. Chen, K. Fu, S. Zhu, W. Luo, Y. Wang, Y. Li, E. Hitz, Y. Yao, J. Dai, J. Wan, V. A. Danner, T. Li, L. Hu, *Nano Lett.* **2016**, *16*, 3616.
[27] A. Milani, *J. Phys. Chem. B* **2015**, *119*, 3868.
[28] B. Saute, R. Narayanan, *Analyst* **2011**, *136*, 527.
[29] B. Hu, D.-W. Sun, H. Pu, Q. Wei, *Talanta* **2020**, *217*, 120998.
[30] J. Trebolazabala, M. Maguregui, H. Morillas, A. de Diego, J. M. Madariaga, *Spectrochimica Acta Part A: Molecular and Biomolecular Spectroscopy* **2017**, *180*, 138.
[31] L. A. F. Dias, E. I. Jussiani, C. R. Appoloni, *J Appl Spectrosc* **2019**, *86*, 166.
[32] J. Sitjar, J.-D. Liao, H. Lee, L. P. Pan, B. H. Liu, W. Fu, G. D. Chen, *Nanomaterials* **2019**, *9*, 1750.
[33] P. Liou, F. X. Nayigiziki, F. Kong, A. Mustapha, M. Lin, *Carbohydrate Polymers* **2017**, *157*, 643.
[34] L. Xiao, S. Feng, M. Z. Hua, X. Lu, *Talanta* **2023**, *254*, 124128.
[35] U. T. Pham, Q. H. T. Phan, L. P. Nguyen, P. D. Luu, T. D. Doan, H. T. Trinh, C. T. Dinh, T. V. Nguyen, T. Q. Tran, D. X. Le, T. N. Pham, T. D. Le, D. T. Nguyen, *Processes* **2022**, *10*, 442.
[36] W. Zhai, M. Cao, Z. Xiao, D. Li, M. Wang, *Foods* **2022**, *11*, 3597.
[37] U. Mogera, H. Guo, M. Namkoong, M. S. Rahman, T. Nguyen, L. Tian, *Sci. Adv.* **2022**, *8*, eabn1736.
[38] H. Ma, Y. Tian, A. Jiao, C. Wang, M. Zhang, L. Zheng, S. Li, M. Chen, *Vibrational Spectroscopy* **2022**, *118*, 103330.
[39] D. Wang, G. Xu, X. Zhang, H. Gong, L. Jiang, G. Sun, Y. Li, G. Liu, Y. Li, S. Yang, X. Liang, *Sensors and Actuators B: Chemical* **2022**, *359*, 131512.
[40] R. Cai, L. Yin, Q. Huang, R. You, S. Feng, Y. Lu, *Nanomaterials* **2022**, *12*, 421.

[41] J. Lin, Y. Weng, X. Lin, S. Qiu, Z. Huang, C. Pan, Y. Li, K. V. Kong, X. Zhang, S. Feng, *Nanomaterials* **2022**, *12*, 2724.
[42] A. Tan, Y. Zhao, K. Sivashanmugan, K. Squire, A. X. Wang, *Food Control* **2019**, *103*, 111.
[43] A. L. Jenkins, R. A. Larsen, T. B. Williams, *Spectrochimica Acta Part A: Molecular and Biomolecular Spectroscopy* **2005**, *61*, 1585.
[44] J. Chen, G. Qin, Q. Chen, J. Yu, S. Li, F. Cao, B. Yang, Y. Ren, *J. Mater. Chem. C* **2015**, *3*, 4933.
[45] R. Michael-Jubeli, A. Tfayli, J. Bleton, A. Baillet-Guffroy, *European Journal of Dermatology* **2011**, *21*, 63.
[46] A. Assi, R. Michael-Jubeli, C. Jacques-Jamin, H. Duplan, A. Baillet-Guffroy, A. Tfayli, *J Raman Spectroscopy* **2023**, *54*, 487.
[47] K. Czamara, K. Majzner, M. Z. Pacia, K. Kochan, A. Kaczor, M. Baranska, *J. Raman Spectrosc.* **2015**, *46*, 4.
[48] V. R. Batistela, J. da Costa Cedran, H. P. Moisés de Oliveira, I. S. Scarminio, L. T. Ueno, A. Eduardo da Hora Machado, N. Hioka, *Dyes and Pigments* **2010**, *86*, 15.
[49] C. Yu, L. Varghese, J. Irudayaraj, *Langmuir* **2007**, *23*, 9114.
[50] A. L. Valle, F. C. C. Mello, R. P. Alves-Balvedi, L. P. Rodrigues, L. R. Goulart, *Environ Chem Lett* **2019**, *17*, 291.
[51] M. M. Peixoto, G. F. Bauerfeldt, M. H. Herbst, M. S. Pereira, C. O. da Silva, *J. Phys. Chem. A* **2015**, *119*, 5241.
