# Supplementary material for: Correction to “Flexible 3D Plasmonic Web Enables Remote Surface Enhanced Raman Spectroscopy”
Source: Adv Sci (Weinh). 2024 Nov 4;12(1):2412492. doi: 10.1002/advs.202412492 (PMC11714189; doi:10.1002/advs.202412492)
Supplement: Supplementary file 1 — Supporting Information [file ADVS-12-2412492-s001.docx]

**Supporting Information**

**Figure S15** (page 19 of the Supporting Information) needs a correction according to the updated Figure below


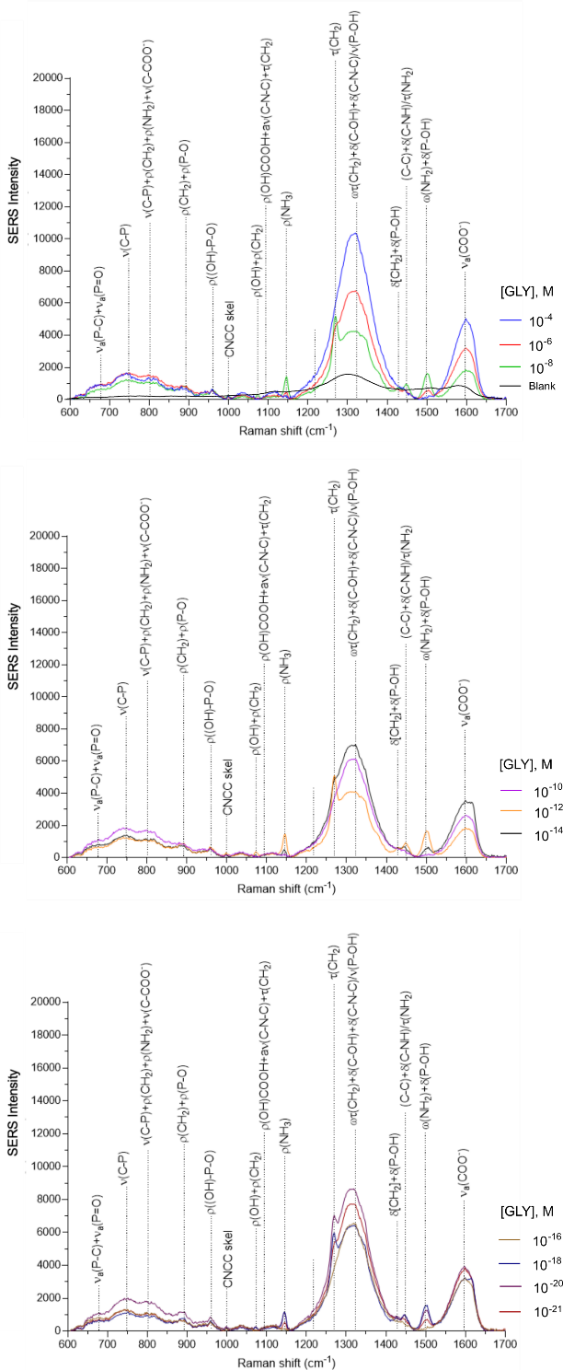


**Figure S15**. Detailed SERS spectra of GLY analyzed at different concentrations using 3D-POWER (BC/GO35/AuNRs). Each spectrum represents the mean of fifty spectra recorded on 2500 μm2 of the corresponding SERS substrate. The spectra were obtained through: excitation wavelength, 785 nm; laser power, 0.08 mW; size of the spot, 1.2 μm; exposure time, 2 s and number of acquisitions, 10.

**Table S10** (page 36 of the Supporting Information) needs a correction according to the updated table below

| **Table S10.** Estimation of the number of GLY molecules. Experimental concentration, (mol/L) | Moles number | Molecules number |
| --- | --- | --- |
| 5.88E-03 | 5.88E-06 | 4E+18 |
| 5.88E-04 | 5.88E-07 | 4E+17 |
| 5.88E-05 | 5.88E-08 | 4E+16 |
| 5.88E-06 | 5.88E-09 | 4E+15 |
| 5.88E-07 | 5.88E-10 | 4E+14 |
| 5.88E-08 | 5.88E-11 | 4E+13 |
| 5.88E-09 | 5.88E-12 | 4E+12 |
| 5.88E-10 | 5.88E-13 | 4E+11 |
| 5.88E-11 | 5.88E-14 | 4E+10 |
| 5.88E-12 | 5.88E-15 | 4E+09 |
| 5.88E-13 | 5.88E-16 | 4E+08 |
| 5.88E-14 | 5.88E-17 | 4E+07 |
| 5.88E-15 | 5.88E-18 | 4E+06 |
| 5.88E-16 | 5.88E-19 | 4E+05 |
| 5.88E-17 | 5.88E-20 | 4E+04 |
| 5.88E-18 | 5.88E-21 | 4E+03 |
| 5.88E-19 | 5.88E-22 | 354.09 |
| 5.88E-20 | 5.88E-23 | 35.41 |
| 5.88E-21 | 5.88E-24 | 3.54 |

SERS substrate: BC/AuNRs or BC/GO35/AuNRs, BC, bacterial nanocellulose; GO, graphene oxide; AuNRs, gold nanorods, [GLY], concentration of glyphosate, M, mol/L.

**Table S12** (page 38 of the Supporting Information) needs a correction according to the updated table below

| **Table S12.** Analytical enhancement factor for GLY via 3D-POWER. [GLY], (M) | | Intensity at Raman shift | | | Molecules number (N) | | | AEF | |
| --- | --- | --- | --- | --- | --- | --- | --- | --- | --- |
| 880 cm-1 | | 970 cm-1 | | | 1417 cm-1 | | | 1500 cm-1 | |
| 5.88E-04 | 811 | | 315 | 1114 | | 150 | 4E+17 | | 3.3E+00 |
| 5.88E-06 | 860 | | 316 | 697 | | 527 | 4E+15 | | 1.1E+03 |
| 5.88E-08 | 796 | | 347 | 571 | | 1641 | 4E+13 | | 3.6E+05 |
| 5.88E-10 | 937 | | 359 | 671 | | 142 | 4E+11 | | 3.1E+06 |
| 5.88E-12 | 820 | | 379 | 418 | | 1669 | 4E+09 | | 3.6E+09 |
| 5.88E-14 | 754 | | 266 | 633 | | 579 | 4E+07 | | 1.3E+11 |
| 5.88E-16 | 662 | | 277 | 734 | | 130 | 4E+05 | | 2.8E+12 |
| 5.88E-18 | 733 | | 236 | 676 | | 1573 | 4E+03 | | 3.4E+15 |
| 5.88E-20 | 1159 | | 440 | 908 | | 1272 | 35.41 | | 2.8E+17 |
| 5.88E-21 | 744 | | 258 | 856 | | 683 | 3.54 | | 1.5E+18 |

BC, bacterial nanocellulose; GO35, graphene oxide at 35 μg·mL-1; AuNRs, gold nanorods, [GLY], concentration of glyphosate, M, mol/L. The AEF, Analytical Enhancement Factor, was obtained through the Raman intensity recorded in BC, GLY was concentrated at 1.8x10-3 M, the Raman intensity at 1500 cm-1 was employed to this end (see the Experimental Section).
